# Decisive gene strategy on osteoarthritis: a comprehensive whole-literature based approach for conclusive gene targets

**DOI:** 10.18632/aging.206094

**Published:** 2024-09-06

**Authors:** Yi-Chou Chen, Yu-Chiao Wang, Meng-Chang Lee, Yu-Hsuan Chen, Wen Su, Pi-Shao Ko, Cheng-Jung Chen, Sui-Lung Su

**Affiliations:** 1 Graduate Institute of Medical Sciences, National Defense Medical Center, Taipei 114201, Taiwan, R.O.C; 2 Department of Orthopedics, Taoyuan General Hospital, Ministry of Health and Welfare, Taoyuan 330, Taiwan, R.O.C; 3 School of Public Health, National Defense Medical Center, Taipei 114201, Taiwan, R.O.C; 4 Graduate Institute of Aerospace and Undersea Medicine, National Defense Medical Center, Taipei 114201, Taiwan, R.O.C; 5 Graduate Institute of Life Sciences, National Defense Medical Center, Taipei 114201, Taiwan, R.O.C; 6 Division of General Surgery, Department of Surgery, Tri-Service General Hospital, National Defense Medical Center, Taipei 114202, Taiwan; 7 Department of Surgery, Chiayi Branch, Taichung Veterans General Hospital, Chiayi City 60090, Taiwan

**Keywords:** osteoarthritis (OA), decisive gene strategy (DGS), trial sequential analysis (TSA)

## Abstract

Background: Previous meta-analyses only examined the association between single or several gene polymorphisms and osteoarthritis (OA), whereas no studies have concluded that there are existing all gene loci that associate with OA.

Objective: To assess whether a definite conclusion of the association between the gene loci and OA can be drawn.

Methods: Decisive gene strategy (DGS), a literature-based approach, was used to search PubMed, Embase, and Cochrane databases for all meta-analyses that associated gene polymorphisms and OA. Trial Sequential Analysis (TSA) examined the sufficiency of the cumulative sample size. Finally, we assessed the importance of gene loci in OA based on whether there were enough sample sizes and the heterogeneity of the literatures with I^2^ value.

Results: After excluding 179 irrelevant publications, 80 meta-analysis papers were recruited. Among Caucasians, SMAD3 rs12901499 (OR = 1.20, 95% CI: 1.12-1.29) was a risk factor with validation of sufficient sample sizes through TSA model. Among Asians, there were 3 gene loci risk factors with validation of sufficient sample sizes through TSA model: ESR1 rs2228480, SMAD3 rs12901499, and MMP-1 rs1799750 (OR = 1.35, 95% CI: 1.08-1.69; OR = 1.34, 95% CI: 1.07-1.69; OR = 1.43, 95% CI: 1.18-1.74, respectively). Besides, 3 gene loci, DVWA rs7639618, GDF5 rs143383, and VDR rs7975232 (OR = 0.78, 95% CI: 0.67-0.90; OR = 0.74, 95% CI: 0.67-0.81; OR = 0.56, 95% CI: 0.35-0.90, respectively) were identified as protective factors through TSA model.

Conclusions: We used DGS to identify conclusive gene loci associated with OA. These findings provide implications of precision medicine in OA and may potentially advance genetic therapy.

## INTRODUCTION

Osteoarthritis (OA) is the most prevalent joint disease worldwide and the leading cause of disability in the older adults [1]. Studies have reported that OA is subjective to genetic risk factors, such as single nucleotide polymorphisms (SNPs) [2, 3]. For example, DVWA rs7639618 and GDF5 rs143383 are susceptible to OA [4, 5]. However, some published studies showed inconsistent results, such as ESR1 rs2228480 and VDR rs7975232 [6, 7].

In the genetic epidemiology, numerous genetic research papers about the association between SNPs and OA have been published [8–13]. However, less unanimous conclusions prevail in this research, as a result, meta-analysis provides the opportunity to integrate related research to obtain relatively or assumedly comprehensive results. Nevertheless, their typical focus on individual genes or gene locations leads to an incomplete portrayal of the intricate interplay between genetic components and diseases. Furthermore, continuous meta-analysis may elevate the risk of Type I errors and misleading results [14–16]. To tackle this challenge, a statistical tool is essential to estimate the optimal cumulative sample size for conducting meta-analyses and to determine the appropriate juncture for concluding the inclusion of new studies. Therefore, a statistical method is necessary to estimate the required cumulative sample size for conducting meta-analyses and to determine when to cease the addition of new studies. TSA addresses this concern by timely halting the continuous accumulation of samples in conventional meta-analysis and employing visual aids to determine the appropriateness of further sample accumulation [17, 18].

In fact, previous meta-analyses on gene polymorphisms and diseases feature two shortcomings: (1) most studies only examined a few genes or loci on a specific disease; (2) the cumulative sample size for the association between a gene/locus and diseases is often overlooked [19]. Therefore, the decisive gene strategy (DGS) has been developed to resolve these two difficulties in osteoporosis (OP) to seek potential risk SNPs of OP which provides potential therapy targets [20]. For example, bisphosphonates (BPs) are the most commonly prescribed medications in patients with OP for long-term treatment. However, around 53% of patients with OP show poor response to BPs therapy, probably due to genetic factors, such as SNPs [21–23]. A case in point, LRP5 is identified as potential OP risk genetic factor in our previous study which may provide a reference of link to BP therapy in the future [20].

DGS is a method to collect whole-literature-based references and to exert trial sequence analysis (TSA) to establish the model to assess the relationships of genes to diseases based on sample sizes. Currently, in the molecular epidemiological study of OA, there have been more than 80 meta-analysis studies exploring the correlation between SNPs and OA, and the results of the studies are often inconsistent. In addition, sample size plays a crucial role in comprehensive analysis, but previous studies rarely took sample size into consideration. Therefore, conclusions are determined by calculating sample sizes using the TSA statistical method which is termed the DGS method to integrate these research results. Based on previous discovery derived from the usage of DGS on OP, this study aimed to use DGS to judge the contradictory association between genes and OA to be fundamental pioneers for precision medicine.

## MATERIALS AND METHODS

### Decisive gene strategy (DGS)

DGS, which exerts keyword search and TSA approaches has developed and applied to OP [20]. DGS resolves pending conclusions derived from the unconfirmed cumulative sample size, often inadvertently overlooked, which is a hefty factor in the association between genetic variants and diseases [20, 24].

### Keyword search

The keywords contained synonyms of osteoarthritis, gene polymorphism, and meta-analysis which were sought in the PubMed, Embase, and Cochrane databases for meta-analyses that examined the correlation between gene polymorphisms and OA (Supplementary Table 1).

**Figure 1:**
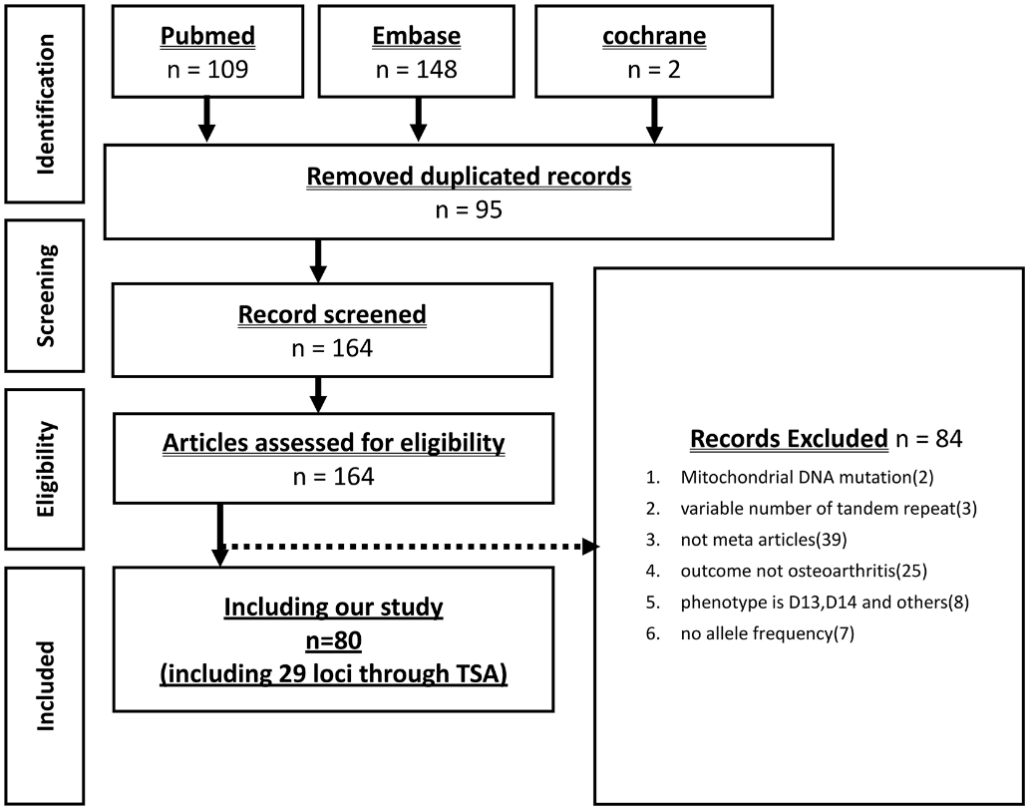
Flow diagram of the identification process for eligible studies.

A comprehensive search was conducted to identify meta-analysis papers, resulting in the discovery of 259 papers from the PubMed, Embase, and Cochrane databases. After excluding 164 papers that overlapped or did not meet the criteria for meta-analysis studies, a total of 80 papers were included in the study, which encompassed 29 SNPs. The literature search process is depicted in Figure 1.

Following the initial keyword search, relevant information including the first author, publication year, refSNP (rs) number, and numbers of case and control groups, as well as the genotype distributions, were extracted.

### Trial sequential analysis (TSA)

Sample sizes from elected papers for OA were integrated into the TSA and analyzed to calculate the required information size (RIS) and to provide the monitoring and futility boundaries for hypothesis testing [25]. This RIS served as a pivotal parameter for TSA, delineating the thresholds for monitoring and futility during hypothesis testing [25]. The statistical validation results of TSA tended to be stable only when the study’s cumulative sample size reached the RIS or when the Z curve in hypothesis testing intersected with the monitoring or futility boundary.

The principle of TSA is to consider meta-analysis as multiple tests, and one additional test is performed every time a new study sample is added. TSA can be employed to correct inflated P-values caused by multiple tests and to decrease the type I error’s occurrence risk. Additionally, TSA formulates two curves for the cumulative sample size: the O’Brien–Fleming and invalid boundaries. The O’Brien–Fleming boundary is plotted according to the quantitation of random error and heterogeneity of accumulated papers, and the invalid boundary is based on a similar theory. Therefore, the two curves can ensure that significant differences are not due to the study results’ excessive inflation.

### Statistical analysis

As the minor allele frequency (MAF) of Caucasians and Asians is different, stratified analysis was performed based on ethnicity (Caucasian and Asian) in TSA. Regarding sample size estimation, type I error, power, and heterogeneity were set to 0.05, 0.8, and 80%, respectively. A review of previous literature showed that the odds ratio (OR) of gene mutations and OA was approximately 1.5. The 1,000-genome database was used as a reference for the MAFs of various loci, and an allele model was used for inheritance mode analysis. A random-effects model was used to combine the sample sizes of studies.

### Availability of data and materials

All data generated or analyzed during this study are included in this published article.

## RESULTS

Among these 29 SNPs, 23 were studied in Caucasians, and 20 were studied in Asians. Accordingly, these SNPs were stratified by ethnicity for TSA. The results of sample size estimation are presented in Supplementary Figures 1–43.

Supplementary Table 2 presents the cumulative sample sizes for 23 SNPs in the Caucasian population. For the 17 gene loci ESR1 rs9340799, ESR1 rs2234693, CYP19A1 rs700518, ESR2 rs1256031, DVWA rs7639618, GDF5 rs143383, VDR rs731236, TGF-β rs1982073, IL-6 rs1800795, IL-6 rs1800797, DIO3 rs945006, EDG2 rs10980705, FRZB rs288326, FRZB rs7775, CALM1 rs12885713, FAS rs1800682, and ADAMTS5 rs226794, the cumulative sample sizes were deemed sufficient to conclude that they are not correlated with OA. However, for the remaining five gene loci, namely TNF-α rs1800629, ADAM12 rs3740199, VDR rs1544410, COX2 rs20417, and MMP-1 rs1799750, more cases were required to draw a conclusion. Additionally, SMAD3 rs12901499 was identified as a risk factor with an odds ratio of 1.20 (95% CI: 1.12-1.29) for its correlation with OA.

Supplementary Table 3 presents the cumulative sample sizes for 20 SNPs in the Asian population. For the 8 gene loci ESR1 rs2234693, CYP19A1 rs700518, ADAM12 rs3740199, ACE I/D rs4340, VDRrs731236, TGF-β rs1982073, FAS rs1800682, ADAMTS5 rs226794, the cumulative sample sizes were deemed sufficient to conclude that they are not correlated with osteoarthritis. However, for the 6 gene loci ESR1 rs9340799, TNF-α rs1800629, RHOB rs585017, TXNDC3 rs4720262, LRCH1 rs912428, CALM1 rs12885713, more cases were required to draw a conclusion. Most importantly, sufficient evidence was obtained to conclude in 6 SNPs that are associated with OA. Among these loci, ESR1 rs2228480 (OR: 1.35, 95%CI: 1.08-1.69), SMAD3 rs12901499 (OR: 1.34, 95%CI: 1.07-1.69), and MMP-1 rs1799750 (OR: 1.43, 95%CI: 1.18-1.74) were identified as risk factors for OA. Conversely, DVWA rs7639618 (OR: 0.78, 95%CI: 0.67-0.90), GDF5 rs143383 (OR: 0.74, 95%CI: 0.67-0.81), and VDR rs7975232 (OR: 0.56, 95%CI: 0.35-0.90) was determined to be a protective factor against OA.

## DISCUSSION

In this study, 80 meta-analysis papers were finally recruited. SMAD3 rs12901499 was a risk factor with adequate sample sizes validation using TSA in Caucasians; 3 gene loci risk factors, ESR1 rs2228480, SMAD3 rs12901499, and MMP-1 rs1799750, while 3 gene loci protective factors, DVWA rs7639618, GDF5 rs143383, and VDR rs7975232, for Asians. We used DGS to identify conclusive gene loci associated with OA. These findings provide implications of precision medicine in OA and may potentially advance genetic therapy.

Currently, the field of genetic research heavily relies on GWAS to unravel the intricate interplay between SNPs and diseases. GWAS enables the simultaneous exploration of millions of SNPs. However, the absence of a hypothesis-driven approach poses a challenge in thoroughly investigating potentially significant SNPs within a biological pathway context. This limitation results in the unresolved issue of missing heritability.

To address potential challenges in GWAS studies and meta-analyses, our research group has developed the DGS for screening OP-related gene loci [20]. In this strategy, we integrate meta-analysis papers and evaluate sample sizes using TSA to statistically analyze whether there are adequate cumulative sample sizes to judge the association between every specific SNP and OA. Through the application of DGS, we identified six gene loci polymorphisms in the Asian population associated with OA.

With validation of sufficient sample sizes through TSA model, there are three risky gene loci/SNPs, ESR1 rs2228480, SMAD3 rs12901499, and MMP-1 rs1799750, for OA; meanwhile, there are three protective gene loci/SNPs, DVWA rs7639618, GDF5 rs143383, and VDR rs7975232, for OA.

ESR1 (also known as ER alpha) belongs to the nuclear receptor superfamily of ligand-regulated transcription factors. ESR1 serves as a vital mediator of signal transduction, and its protein expression is present in cells of the musculoskeletal system, including bone cells and chondrocytes [26]. A recent study demonstrates a new estrogen-independent role of ESR1 in mediating chondrocyte phenotype and its response to mechanical loading and suggests that enhancing ESR1 level may represent a new method to treat OA [27]. Three restriction enzymes defined variants of the ESR1 gene which have been studied in numerous studies, including rs2228480, rs9340799, and rs2234693. Previous study indicated that ESR1 rs2228480 and rs9340799 rather than rs2234693 polymorphisms are associated with the incidence of OA [6]. Similar to our TSA result, ESR1 rs2228480 is conclusively and hazardously associated with OA in Asians; ESR1 rs9340799 is conclusively not associated with OA in Caucasians while more samples are needed to come to conclusions in Asians; ESR1 rs2234693 is conclusively not associated with OA and suggested no necessity of future studies in all populations.

The SMAD3 (SMAD family member 3) gene, located on chromosome 15q22.33, plays a crucial role in joint homeostasis [28, 29]. It serves as a downstream mediator in the TGF-B (transforming growth factor-b) signaling pathway, which is essential for preserving the integrity of articular cartilage [30]. Within the TGF-β signaling pathway, SMAD3 translocates into the nucleus to regulate target genes transcription, thus influencing the cartilage phenotype through interactions with DNA and transcription factors [31]. Various meta-analysis studies supported that SMAD3 rs12901499 was associated with OA risk [32–35]. In our study, we conclude that the SMAD3 rs12901499 exhibits a significant association with susceptibility to OA in both Caucasian and Asian populations.

MMP-1, a significant member of the matrix metalloproteinase (MMPs) family, plays a crucial role in the degradation and deterioration of articular cartilage and bone. It is closely linked to OA, periodontal disease, rheumatoid arthritis, and certain tumors [36–38]. A published meta-analysis indicated that MMP-1 rs1799750 has lack of association with OA susceptibility in all populations [39]. Another three published meta-analyses suggested that this polymorphism may be associated with the risk of younger OA cases [40–42]. According to our TSA results, MMP-1 rs1799750 is concluded as a risk factor for OA in Asians but more sample sizes are needed to determine its association with OA in Caucasians.

The DVWA gene, alternatively referred to as COL6A4P1, exhibits specific expression in cartilage and encodes a protein containing double von Willebrand factor A domains (VWA domain). This protein is involved in cellular adhesion and protein-to-protein interactions [43]. Through DGS analysis, we found a protective effect of DVWA rs7639618 in Asians, while there was no correlation in Caucasians. These findings are consistent with previous research [44–46].

GDF5 is an extracellular signaling molecule and a member of the TGF- superfamily. It participates in the development, maintenance, and repair of synovial joint tissues [47, 48]. GDF5 rs143383 is one of the most investigated polymorphisms in OA studies [47–49]. In all populations, previous meta-analyses of the GDF5 rs143383 polymorphism was found to provide protection for knee OA occurrence [48–50]. In our study, TSA results show GDF5 rs143383 is a protective factor for OA in Asians; however, more sample sizes are needed to determine its association with OA in Caucasians.

Vitamin D has been demonstrated to promote proteoglycan synthesis by facilitating *in vitro* maturation of articular cartilage, suggesting a direct impact on articular cartilage metabolism [51]. Considering the significance of the vitamin D system in bone development and the nature of osteophytes [52], abnormalities in the vitamin D receptor (VDR) gene have been considered potential contributors to OA. Among VDR polymorphisms, rs154410, rs731236, and rs7975232 have been the most extensively studied in relation to OA. However, the association of VDR gene polymorphisms with OA remains controversial [53–55]. Besides, ethnic stratification also provides no evidence to support an association between VDR gene polymorphisms and OA in Caucasians or Asians. Our DGS indicated that VDR rs731236 is not related to OA in all populations; there should be more samples to conclude in VDR rs1544410; in addition, VDR rs7975232 was concluded as a protective factor for OA.

In this study, DGS is adopted to confirm the association of gene loci and OA. Strengths of DGS, usage of meta-analysis literature search and TSA to identify candidate disease-related genes and to overcome the issue of small sample sizes encountered in traditional genetic association studies, are beneficial to address the challenge of estimating the required number of samples for meta-analysis accumulation. Besides, current advanced technology or future visions for OA treatment contain autologous adipose-derived stem cell cartilage regenerative medicine and CRISPR programming. The loci identified by our DGS could potentially serve as targets for CRISPR-based gene editing therapies for cartilage in the future [56].

While the DGS possesses notable strengths, it also exhibits certain limitations. Firstly, in the initial literature search, the DGS was exclusively applied to meta-analysis papers, potentially missing gene loci not encompassed in such studies and solely examining those already covered in meta-analyses. Secondly, the application of the DGS to review meta-analysis papers was restricted to English language publications, thus excluding non-English sources. Additionally, the scope of the DGS is confined to the analysis of individual genes or loci, preventing it from offering a comprehensive assessment of the correlation between genetic factors and diseases.

## CONCLUSIONS

The innovative DGS presents an opportunity for the identification of potential gene loci linked to OA. As exemplified in this study, we applied this methodology to discover 6 gene loci associated with OA in Asians and one in Caucasians. Moving forward, this approach could potentially be extended to other diseases, enabling the integration of disease-related genes into clinical practice and facilitating the development of effective disease prevention strategies.

## Supplementary Material

Supplementary Figures

Supplementary Table 1

Supplementary Table 2

Supplementary Table 3
