# Supplementary material for: Decisive gene strategy on osteoarthritis: a comprehensive whole-literature based approach for conclusive gene targets
Source: Aging (Albany NY). 2024 Sep 6;16(17):12346–78. doi: 10.18632/aging.206094 (PMC11424587; doi:10.18632/aging.206094)
Supplement: Supplementary Figures [file aging-16-206094-s001.pdf]

SUPPLEMENTARY FIGURES

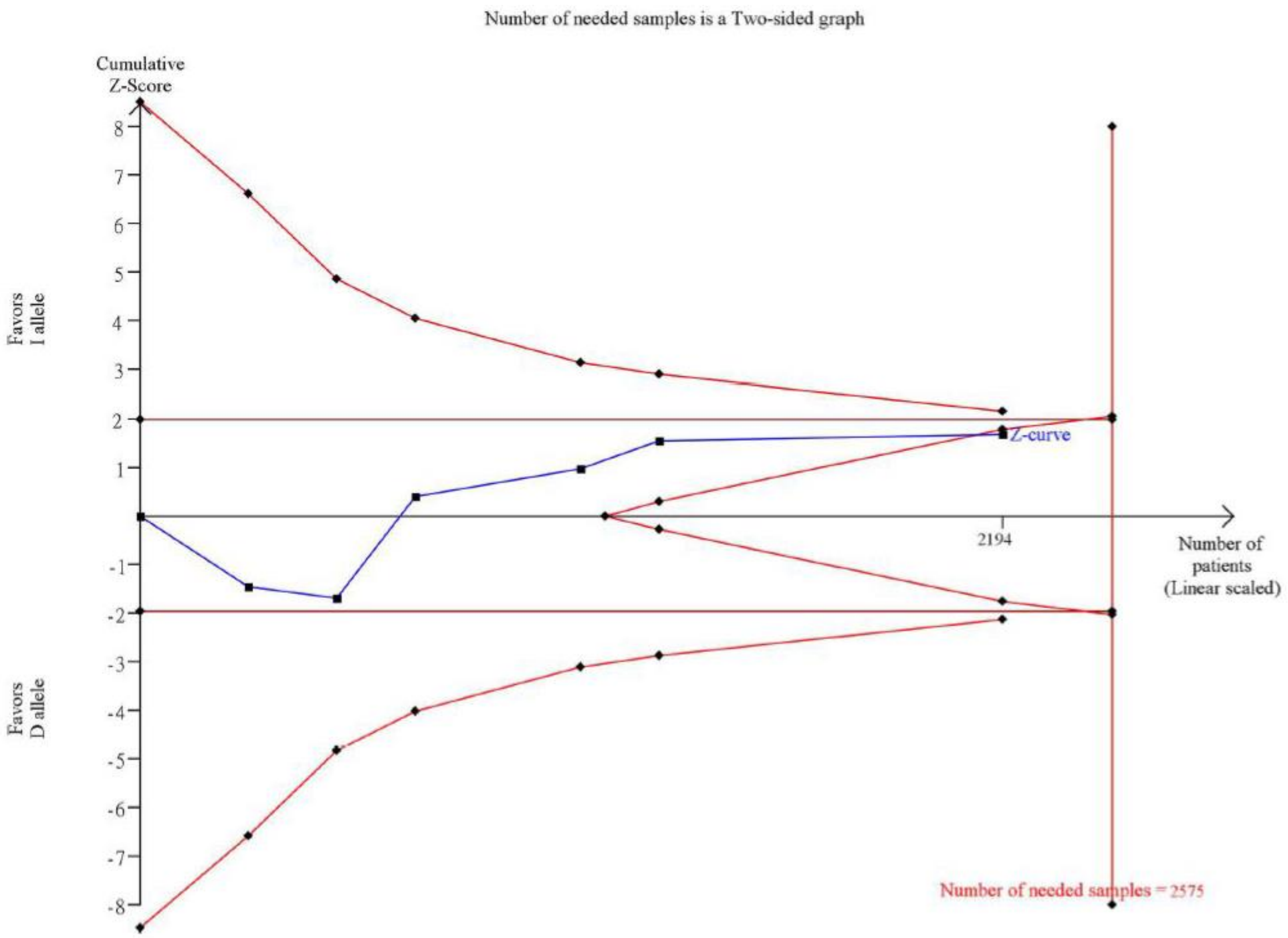

Supplementary Figure 1. Trial sequential analysis of the association between ACE I/D rs4340 polymorphism and the risk of osteoarthritis in Asian.

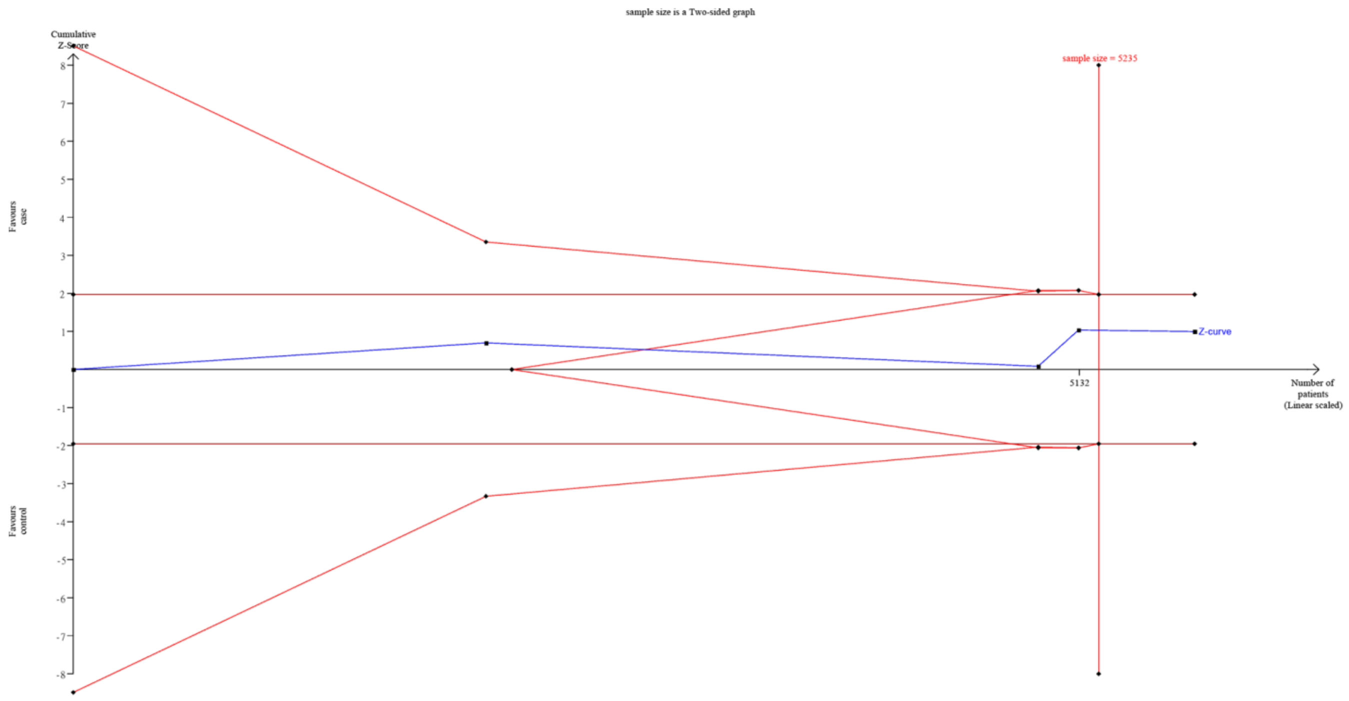

Supplementary Figure 2. Trial sequential analysis of the association between ADAM12 rs3740199 polymorphism and the risk of osteoarthritis in Asian.

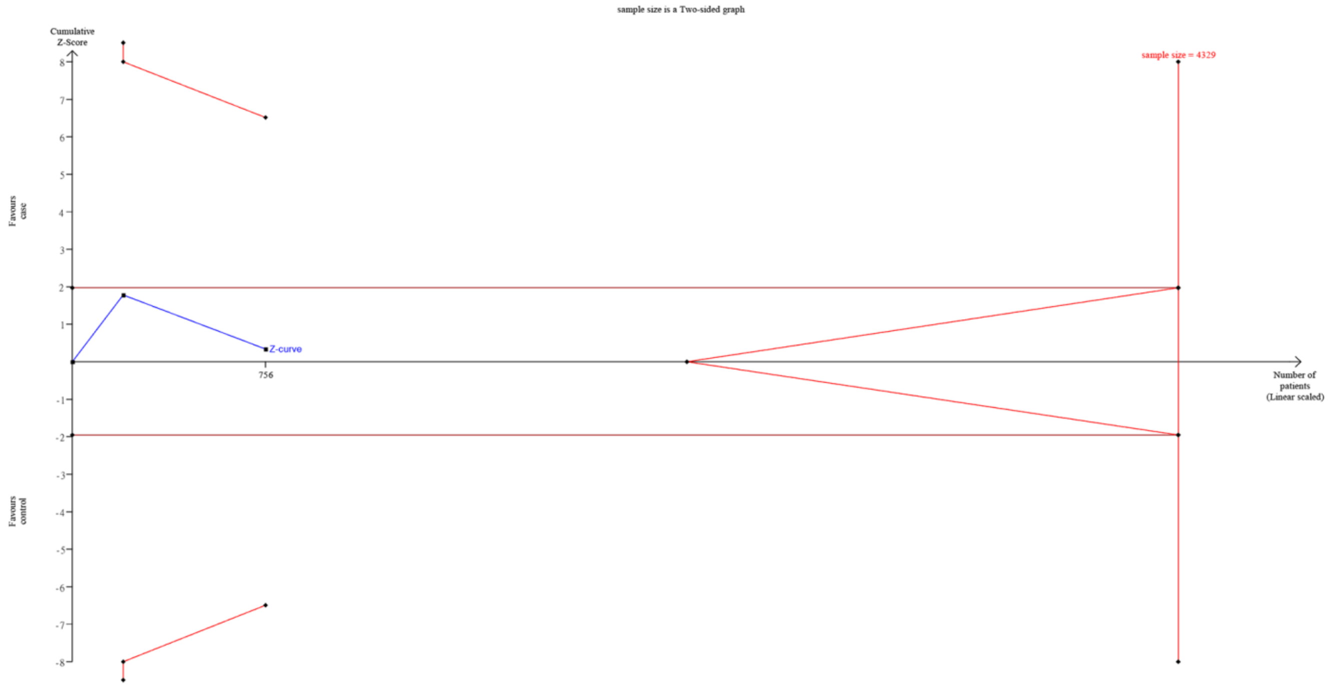

Supplementary Figure 3. Trial sequential analysis of the association between ADAM12 rs3740199 polymorphism and the risk of osteoarthritis in Caucasian.

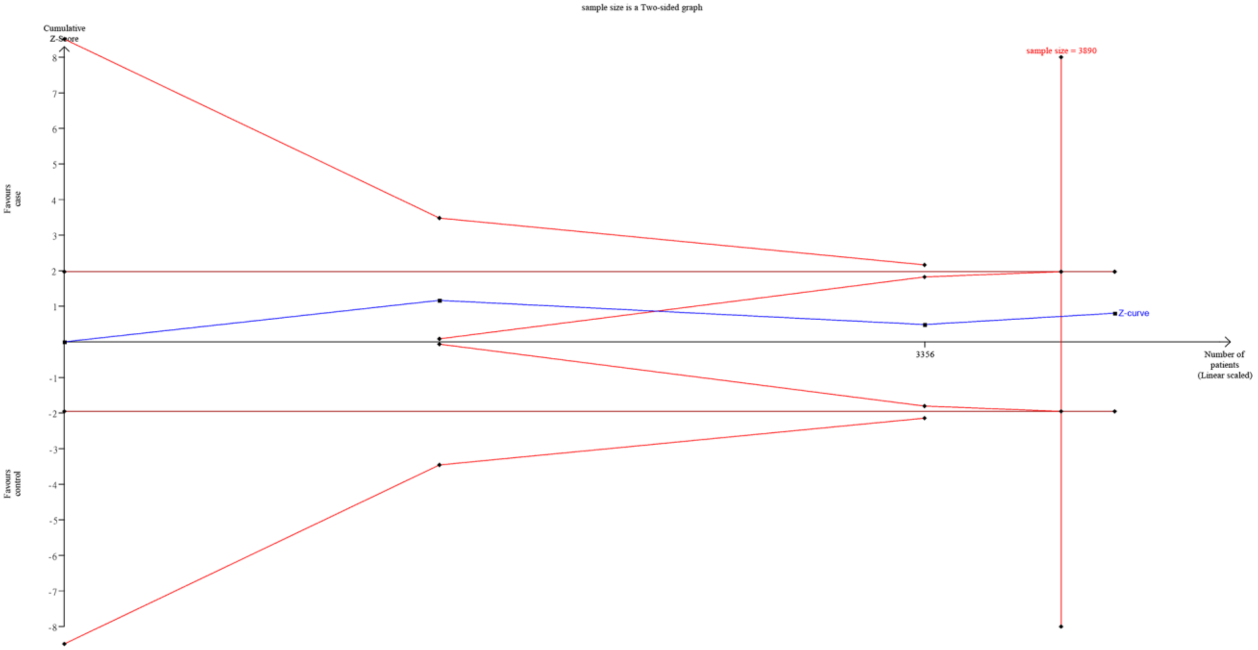

Supplementary Figure 4. Trial sequential analysis of the association between ADAMTS5 rs226794 polymorphism and the risk of osteoarthritis in Asian

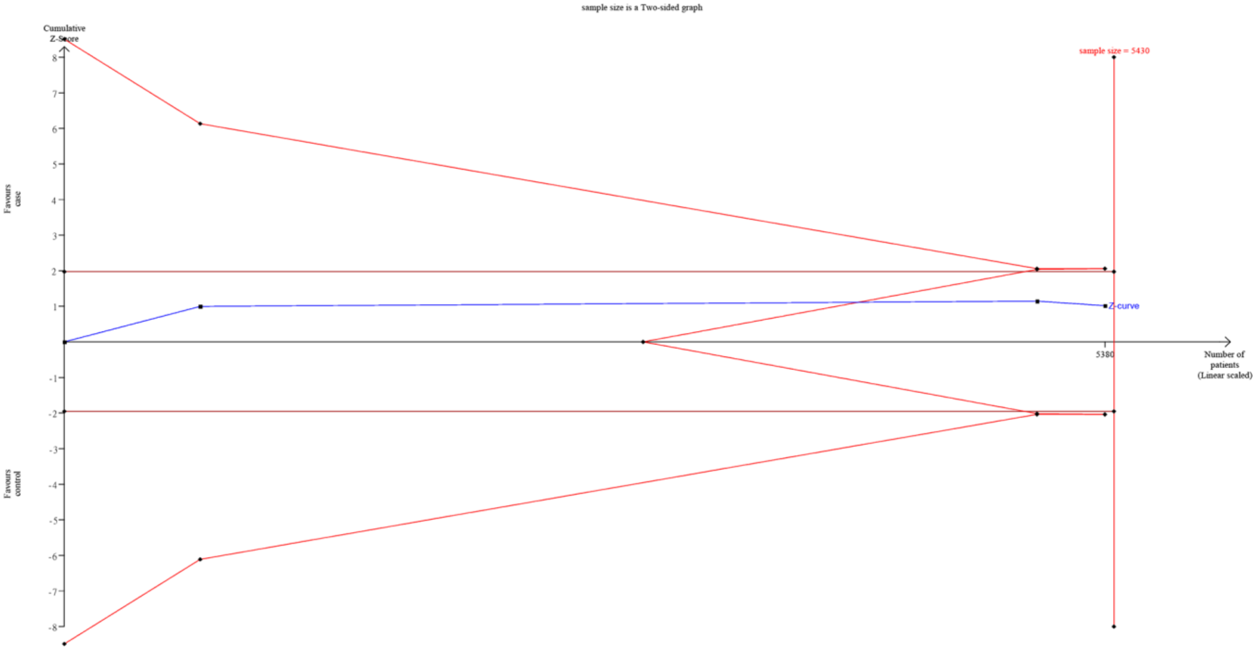

Supplementary Figure 5. Trial sequential analysis of the association between ADAMTS5 rs226794 polymorphism and the risk of osteoarthritis in Caucasian.

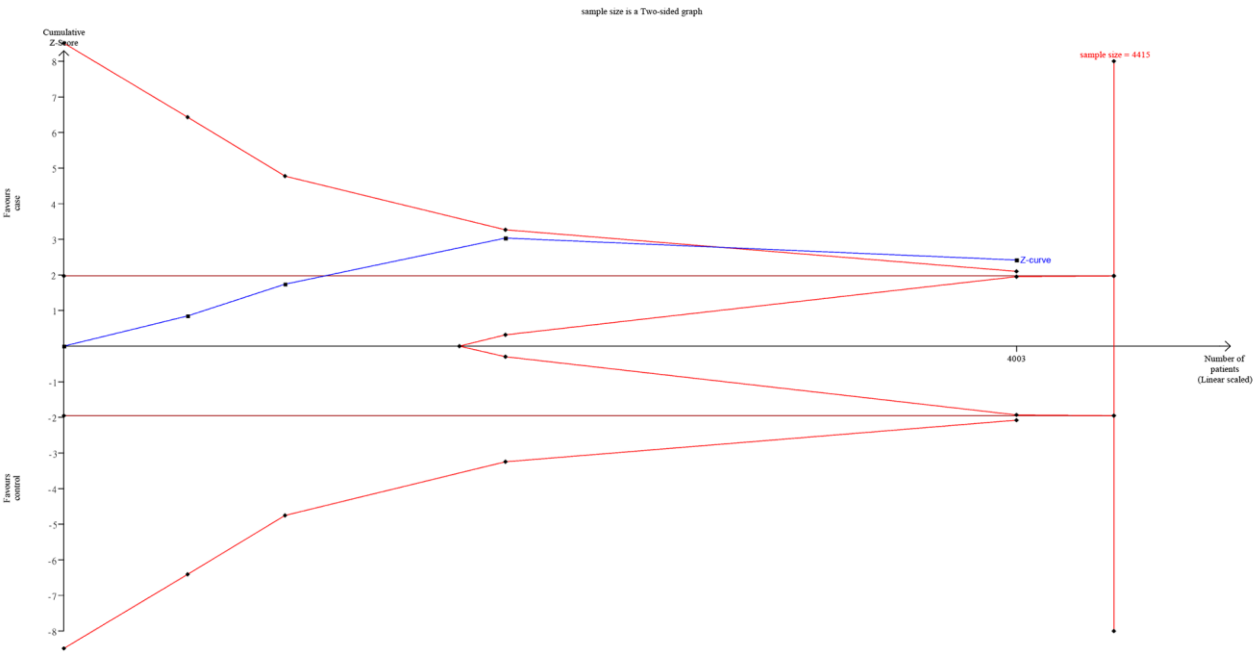

Supplementary Figure 6. Trial sequential analysis of the association between VDR rs7975232 polymorphism and the risk of osteoarthritis in Asian.

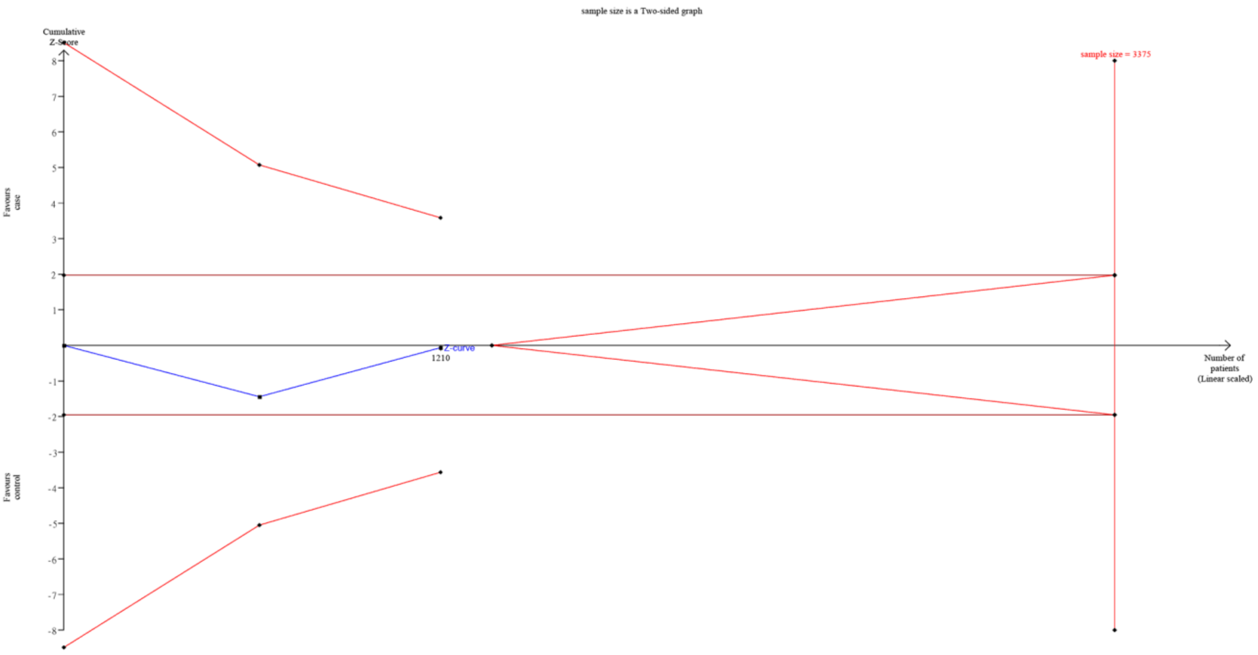

Supplementary Figure 7. Trial sequential analysis of the association between VDR rs1544410 polymorphism and the risk of osteoarthritis in Caucasian.

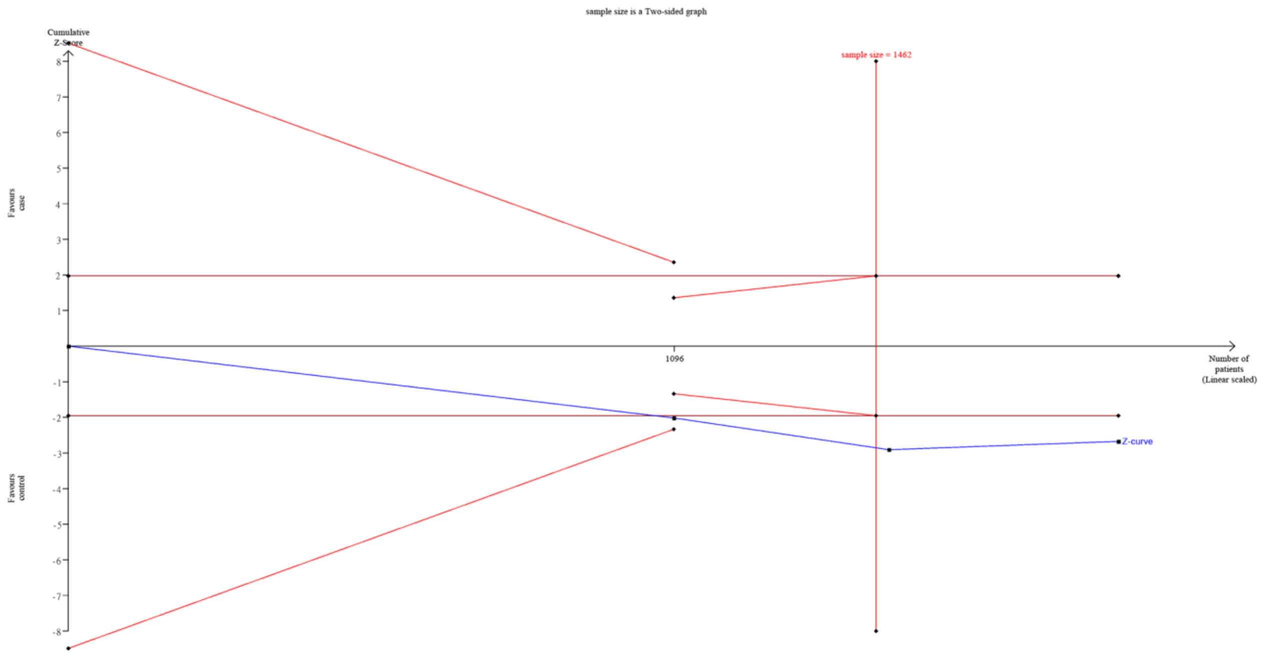

Supplementary Figure 8. Trial sequential analysis of the association between ESR1 rs2228480 polymorphism and the risk of osteoarthritis in Asian.

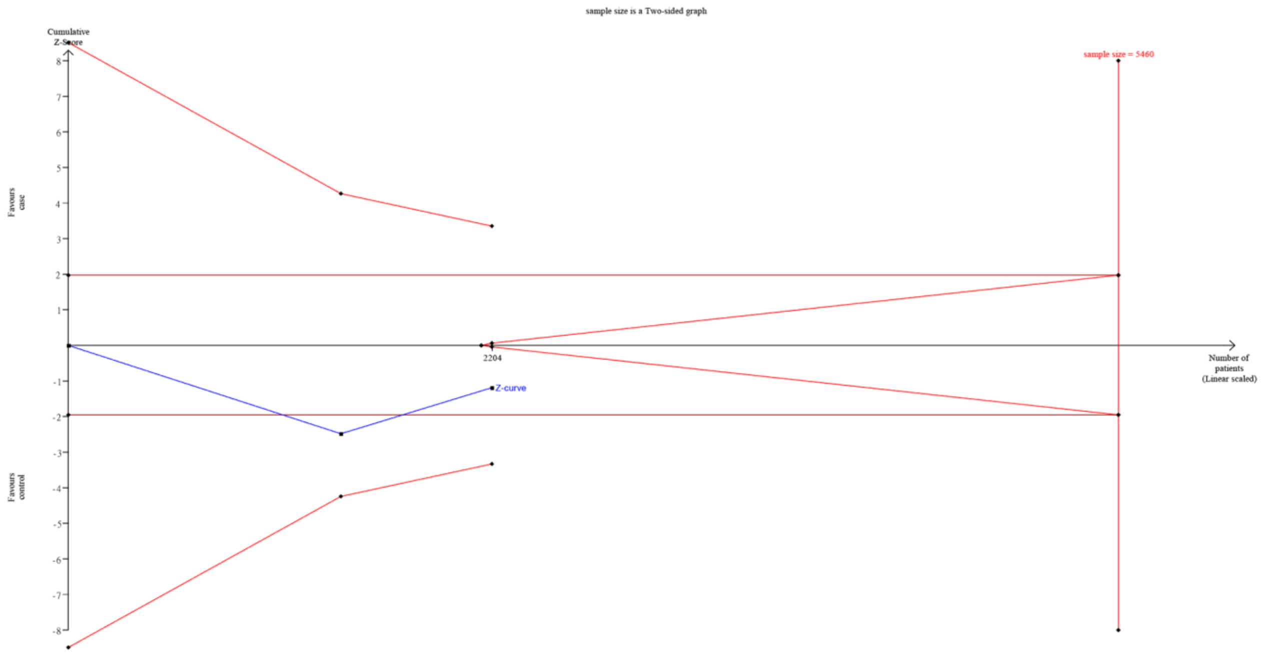

Supplementary Figure 9. Trial sequential analysis of the association between CALM1 rs12885713 polymorphism and the risk of osteoarthritis in Asian.

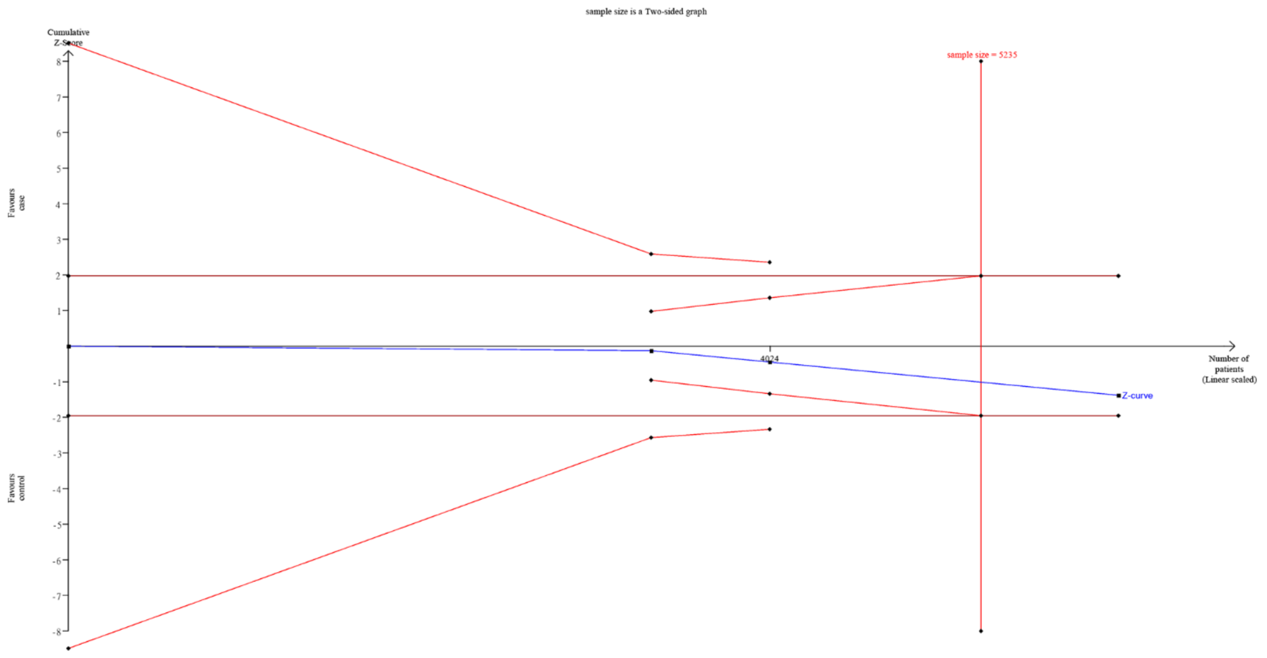

Supplementary Figure 10. Trial sequential analysis of the association between CALM1 rs12885713 polymorphism and the risk of osteoarthritis in Caucasian.

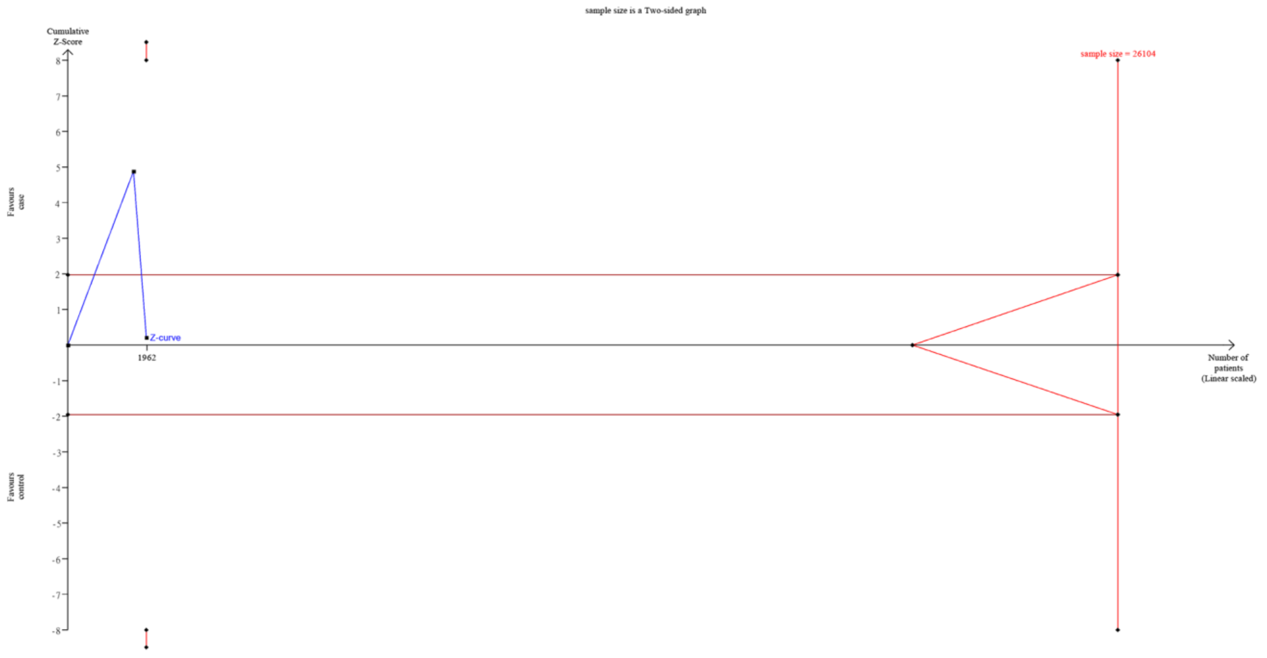

Supplementary Figure 11. Trial sequential analysis of the association between COX2 rs20417 polymorphism and the risk of osteoarthritis in Caucasian.

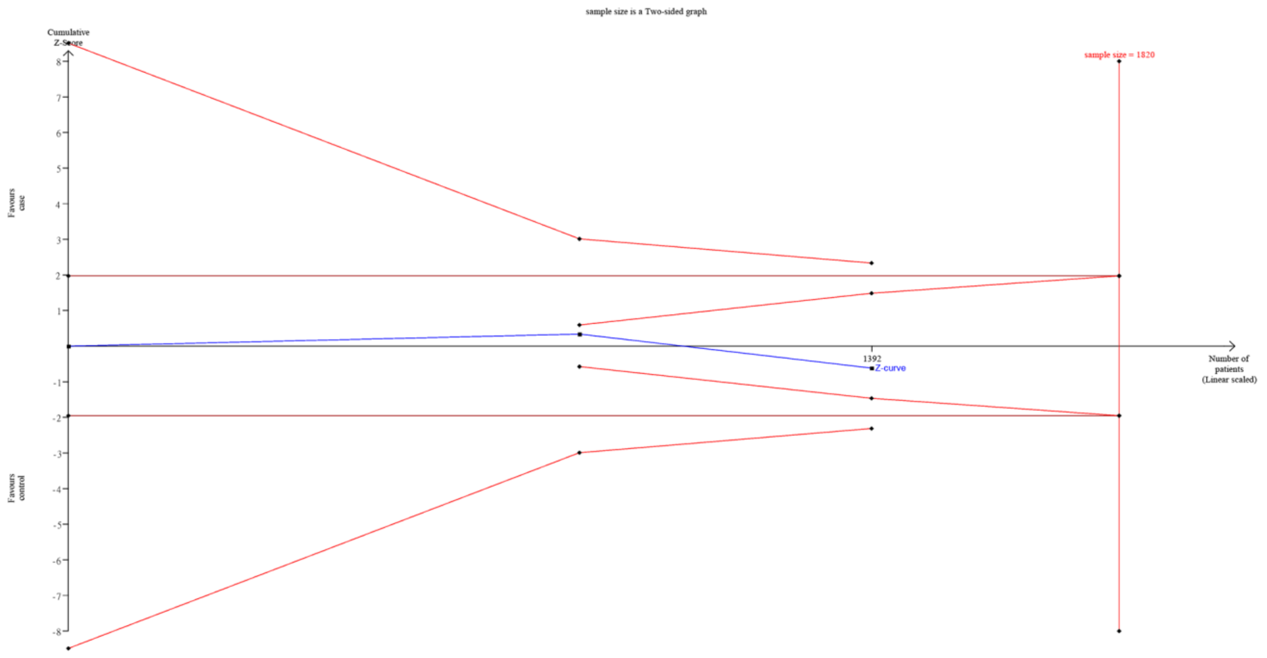

Supplementary Figure 12. Trial sequential analysis of the association between CYP19A1 rs700518 polymorphism and the risk of osteoarthritis in Asian.

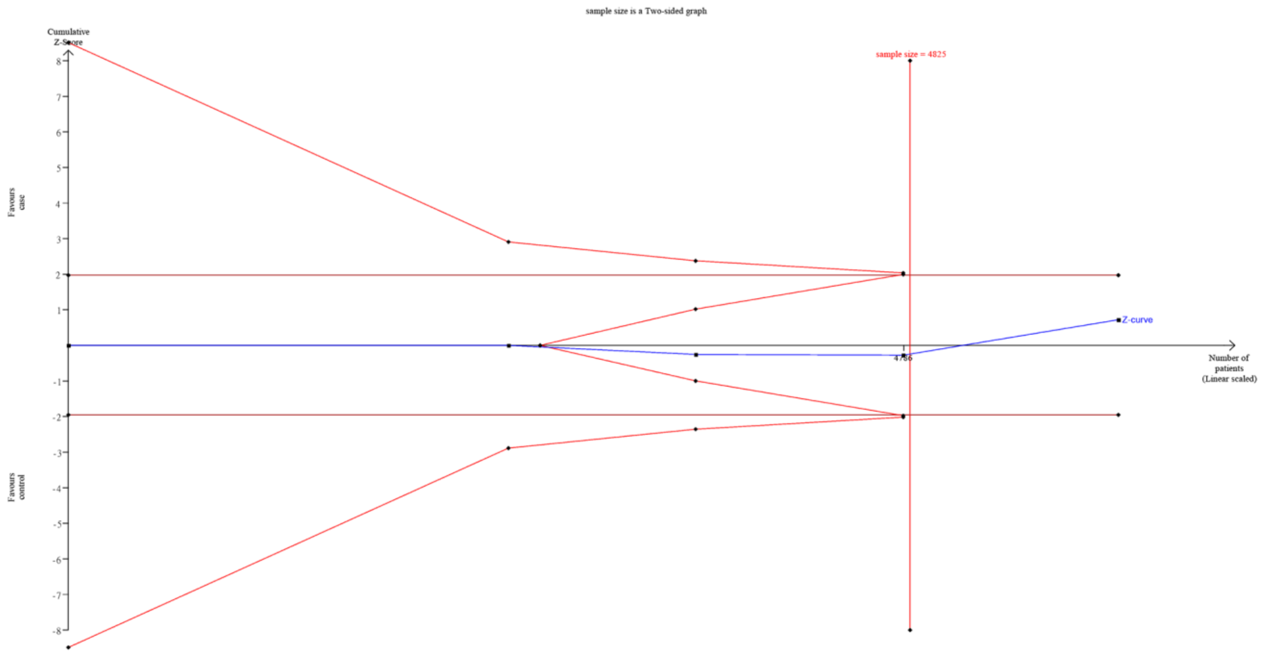

Supplementary Figure 13. Trial sequential analysis of the association between CYP19A1 rs700518 polymorphism and the risk of osteoarthritis in Caucasian.

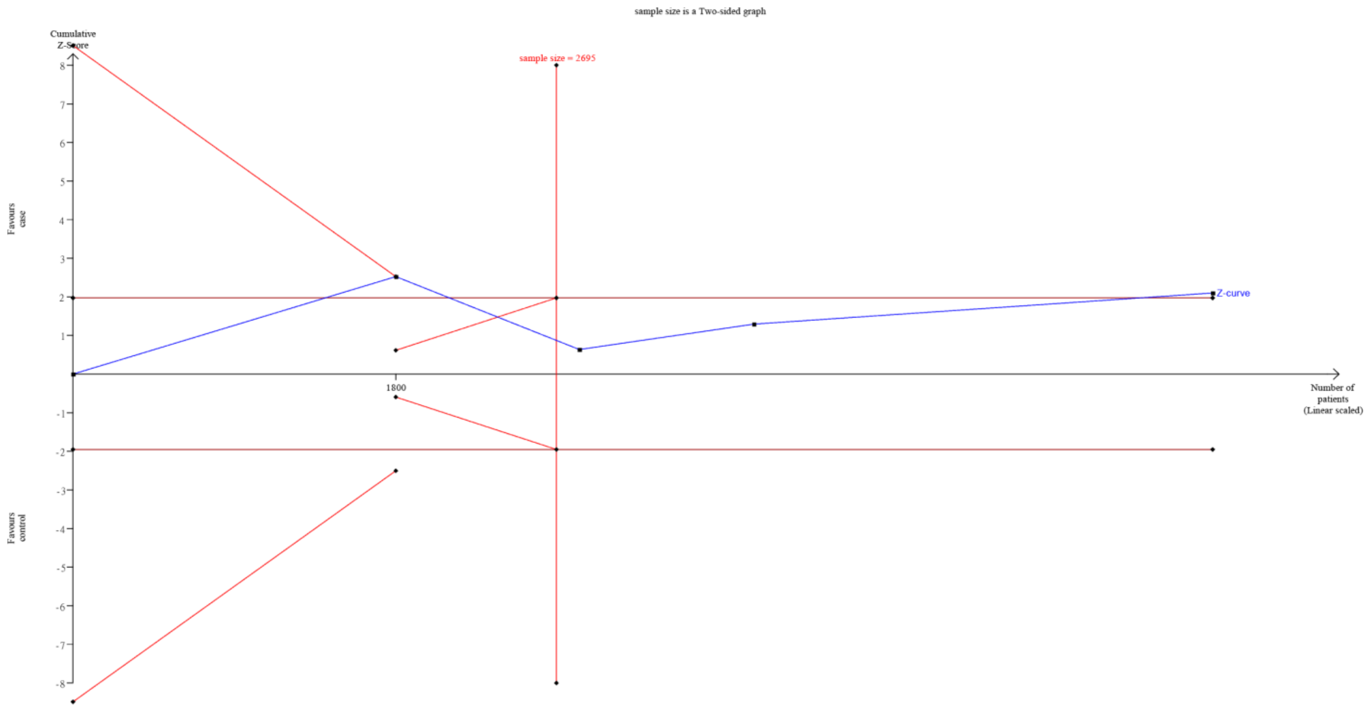

Supplementary Figure 14. Trial sequential analysis of the association between DIO3 rs945006 polymorphism and the risk of osteoarthritis in Caucasian.

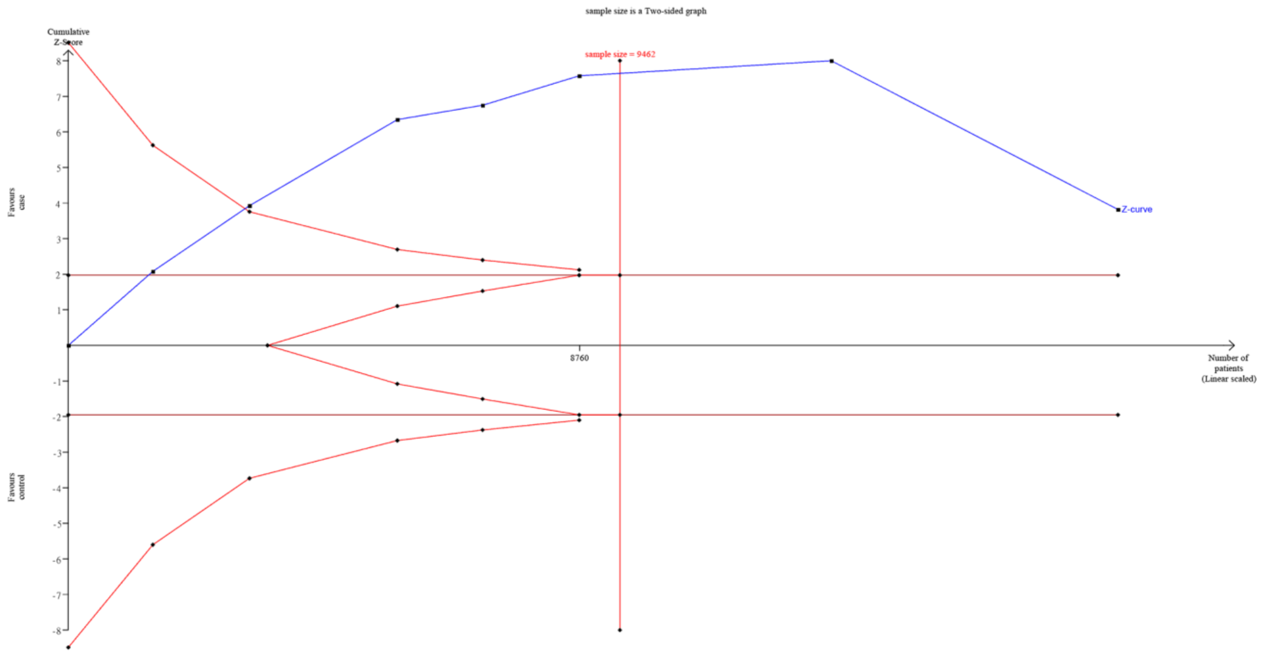

Supplementary Figure 15. Trial sequential analysis of the association between DVWA rs7639618 polymorphism and the risk of osteoarthritis in Asian.

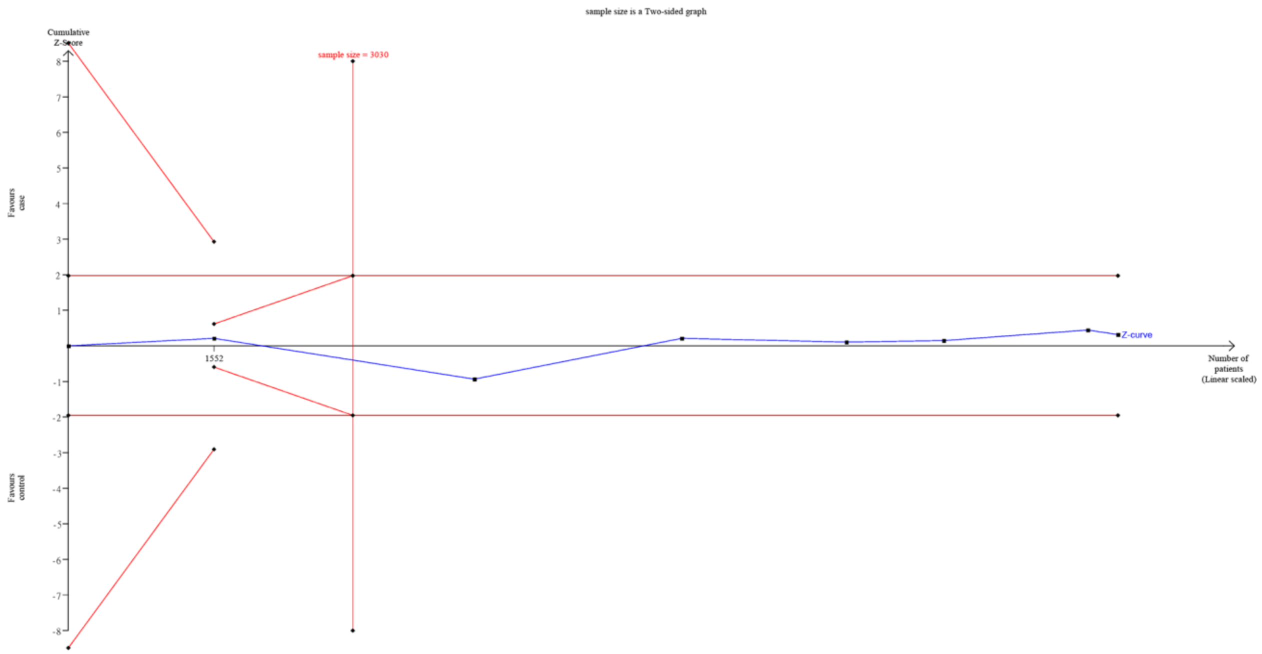

Supplementary Figure 16. Trial sequential analysis of the association between DVWA rs7639618 polymorphism and the risk of osteoarthritis in Caucasian.

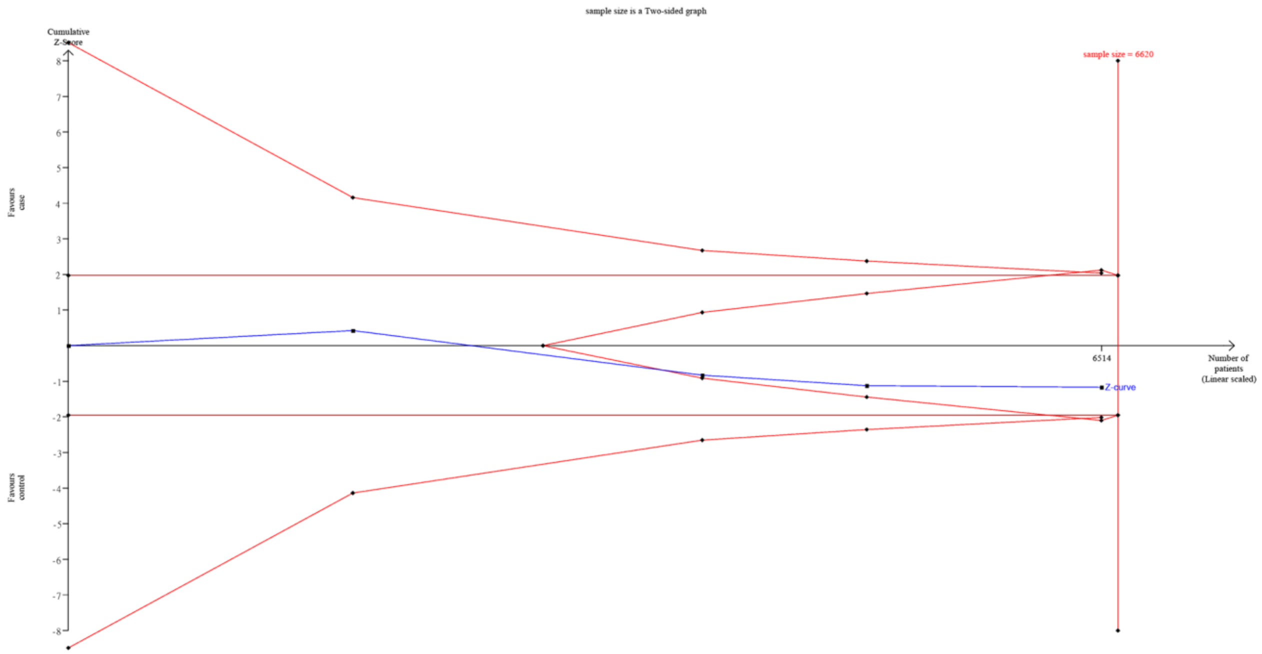

Supplementary Figure 17. Trial sequential analysis of the association between EDG2 rs10980705 polymorphism and the risk of osteoarthritis in Caucasian.

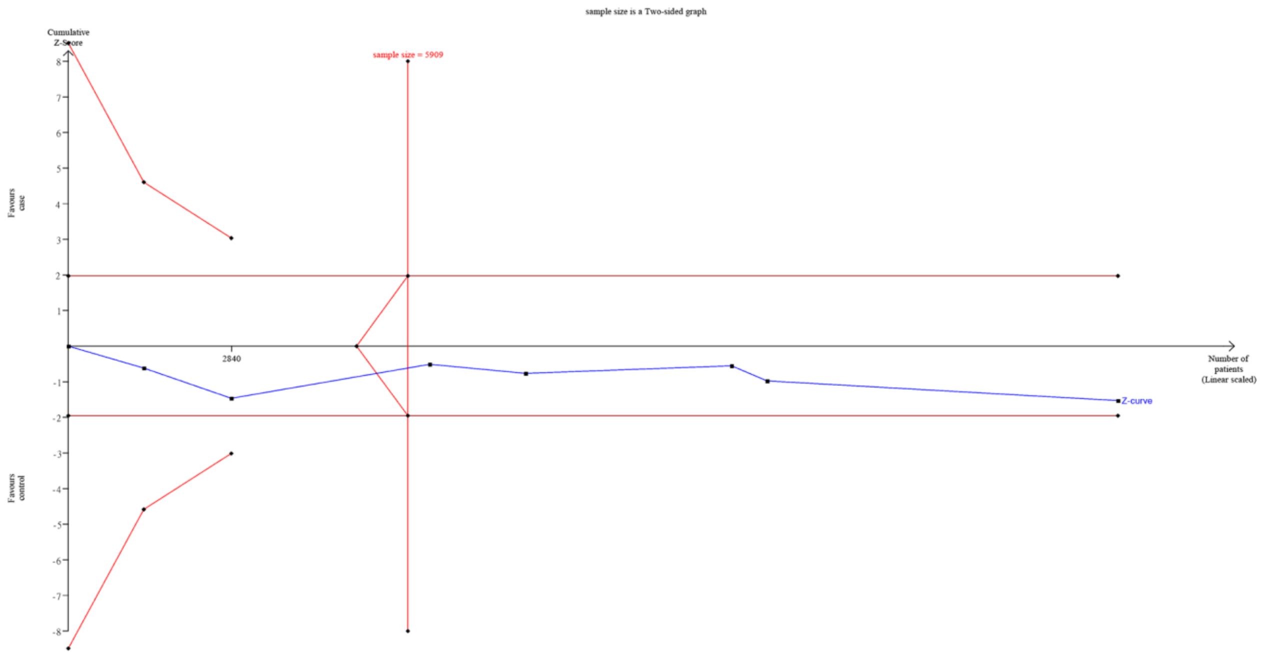

Supplementary Figure 18. Trial sequential analysis of the association between ESR2 rs1256031 polymorphism and the risk of osteoarthritis in Caucasian.

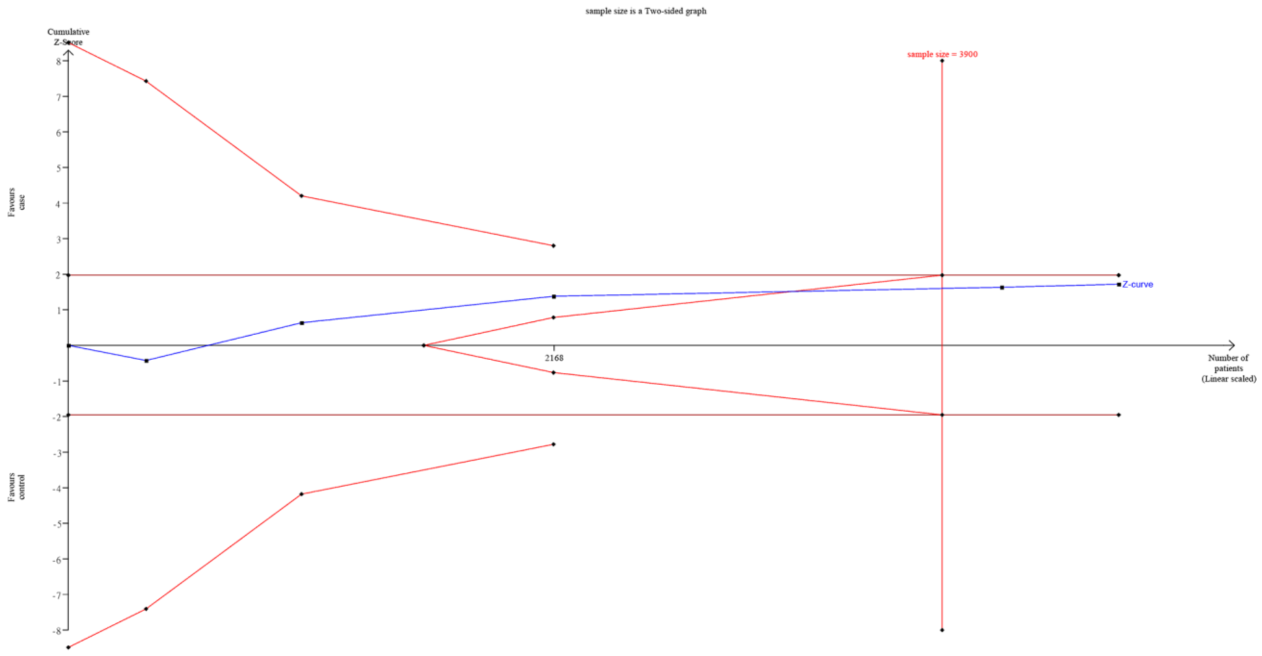

Supplementary Figure 19. Trial sequential analysis of the association between FAS rs1800682 polymorphism and the risk of osteoarthritis in Asian.

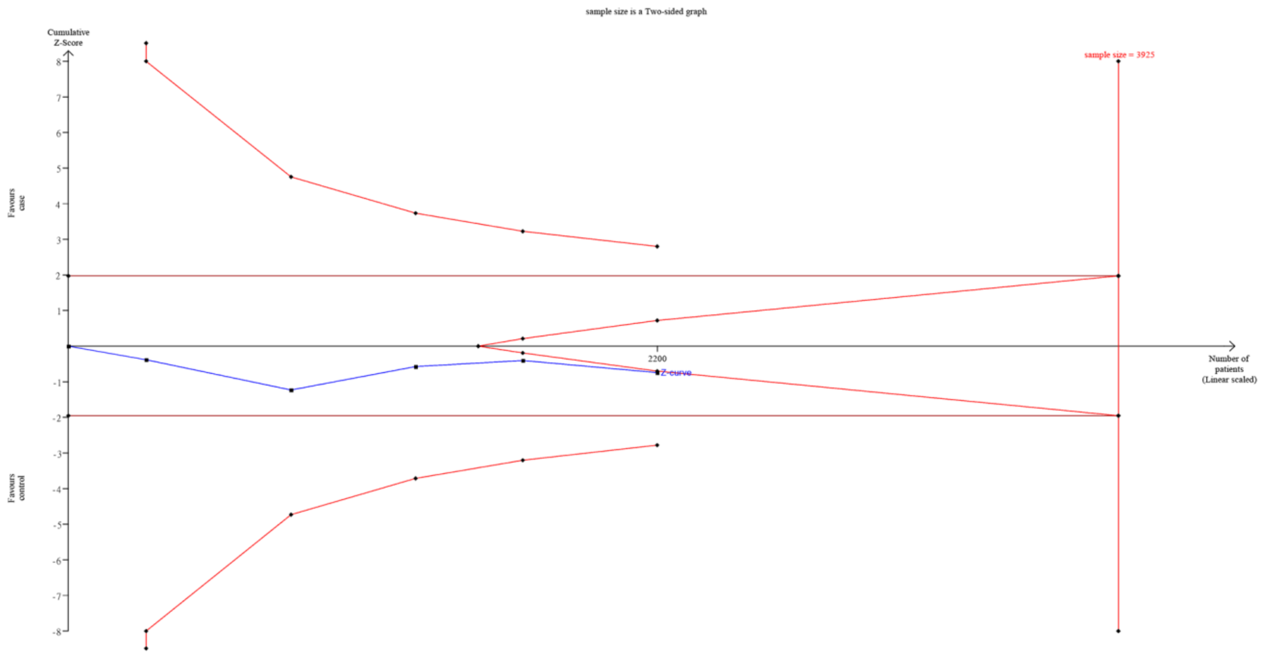

Supplementary Figure 20. Trial sequential analysis of the association between FAS rs1800682 polymorphism and the risk of osteoarthritis in Caucasian.

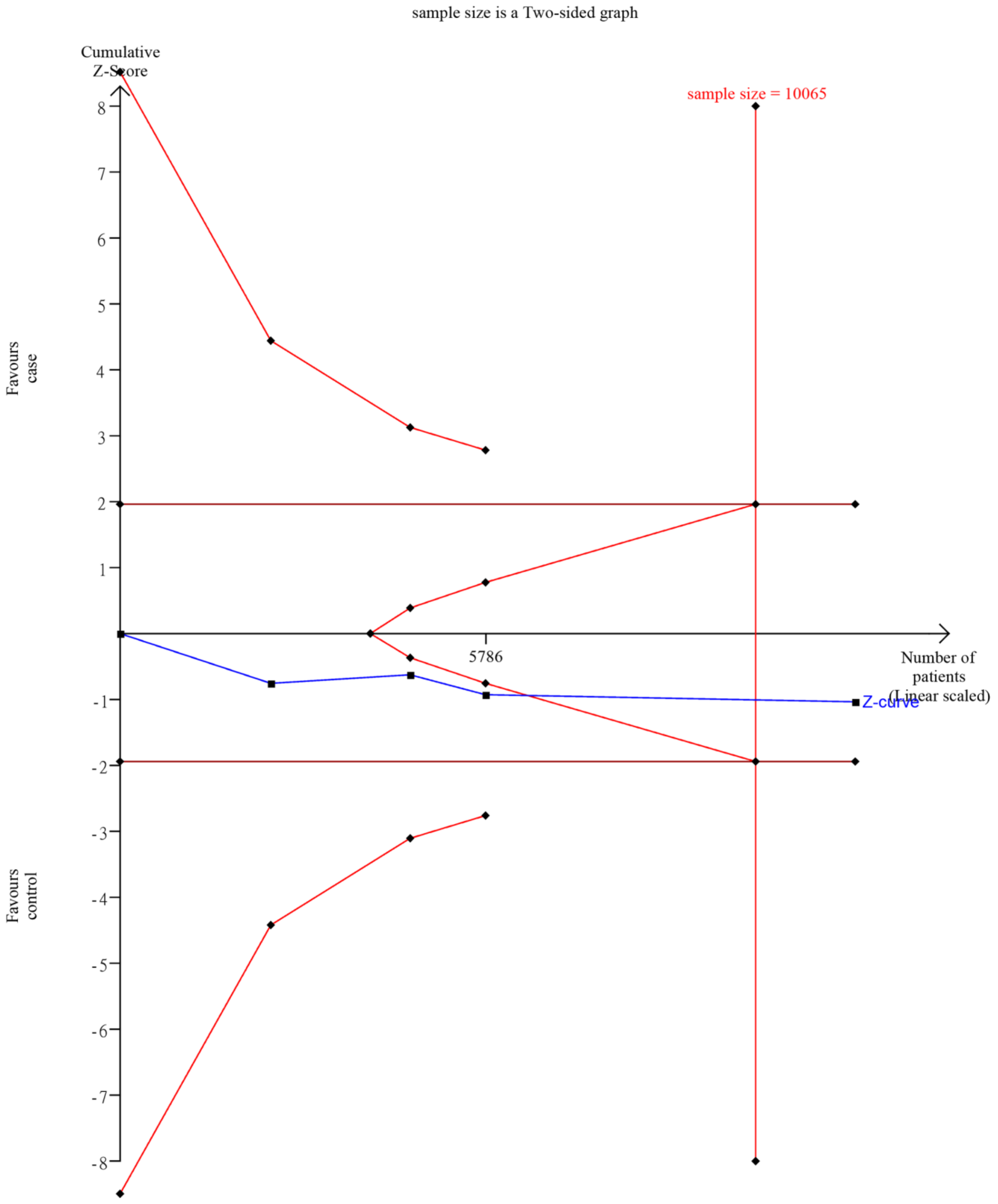

Supplementary Figure 21. Trial sequential analysis of the association between FRZB rs7775 polymorphism and the risk of osteoarthritis in Caucasian.

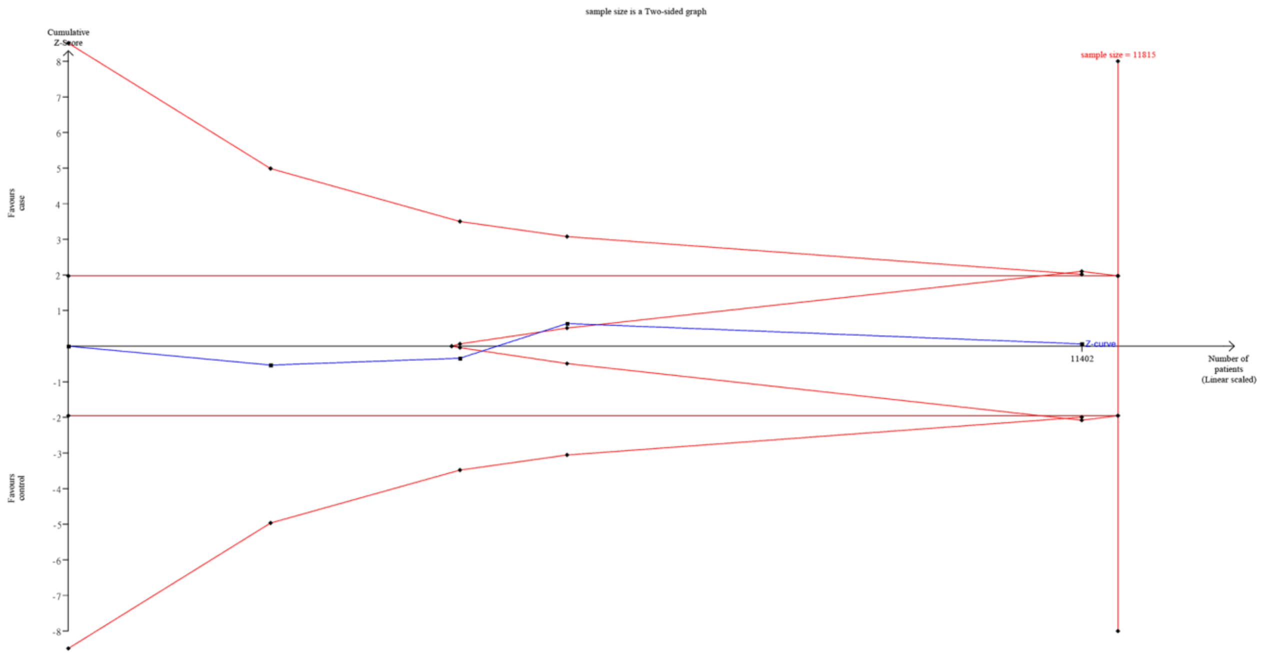

Supplementary Figure 22. Trial sequential analysis of the association between FRZB rs288326 polymorphism and the risk of osteoarthritis in Caucasian.

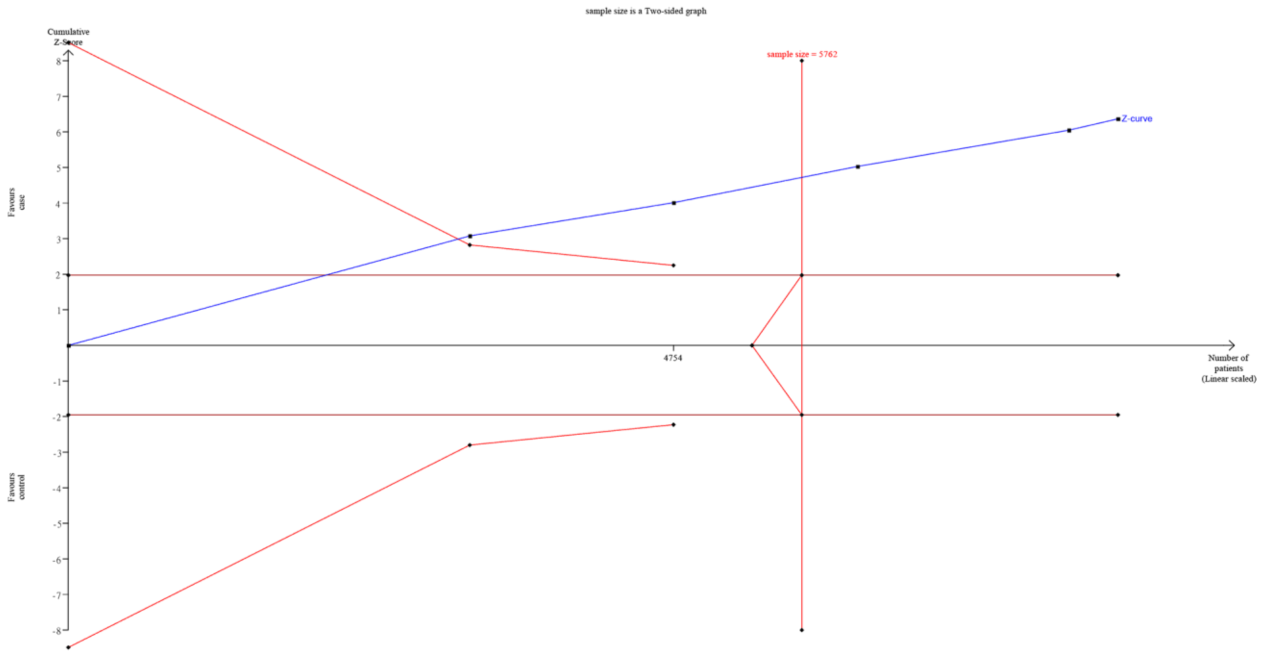

Supplementary Figure 23. Trial sequential analysis of the association between GDF5 rs143383 polymorphism and the risk of osteoarthritis in Asian.

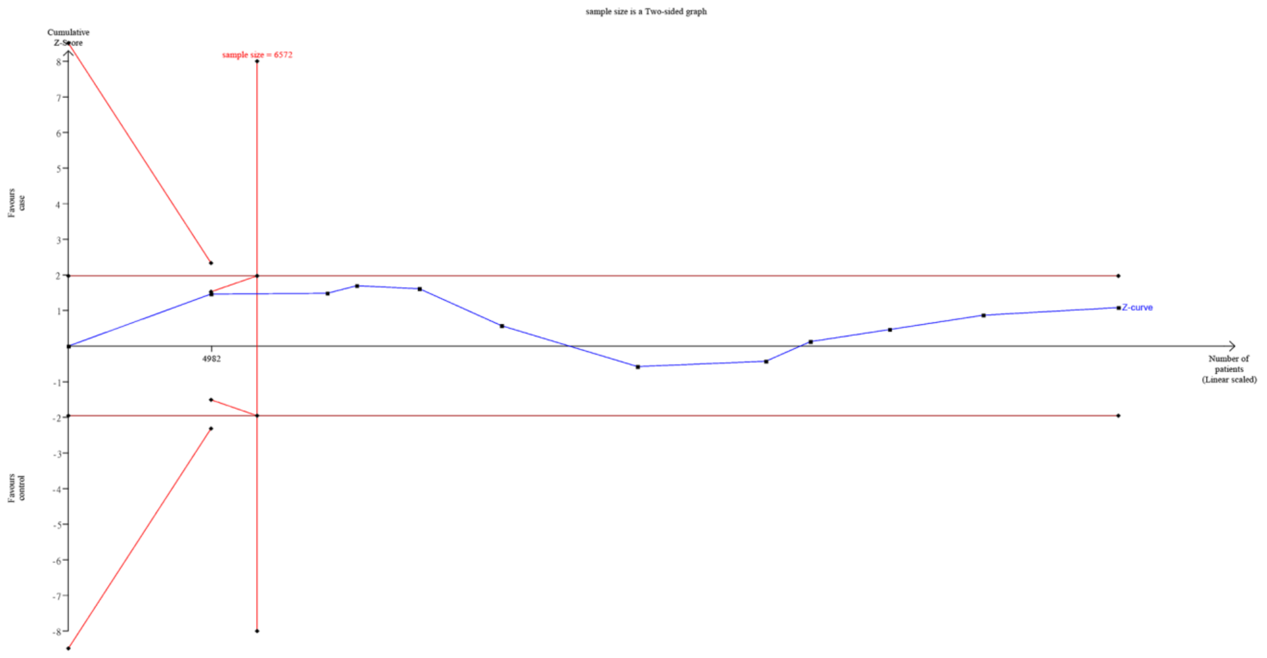

Supplementary Figure 24. Trial sequential analysis of the association between GDF5 rs143383 polymorphism and the risk of osteoarthritis in Caucasian.

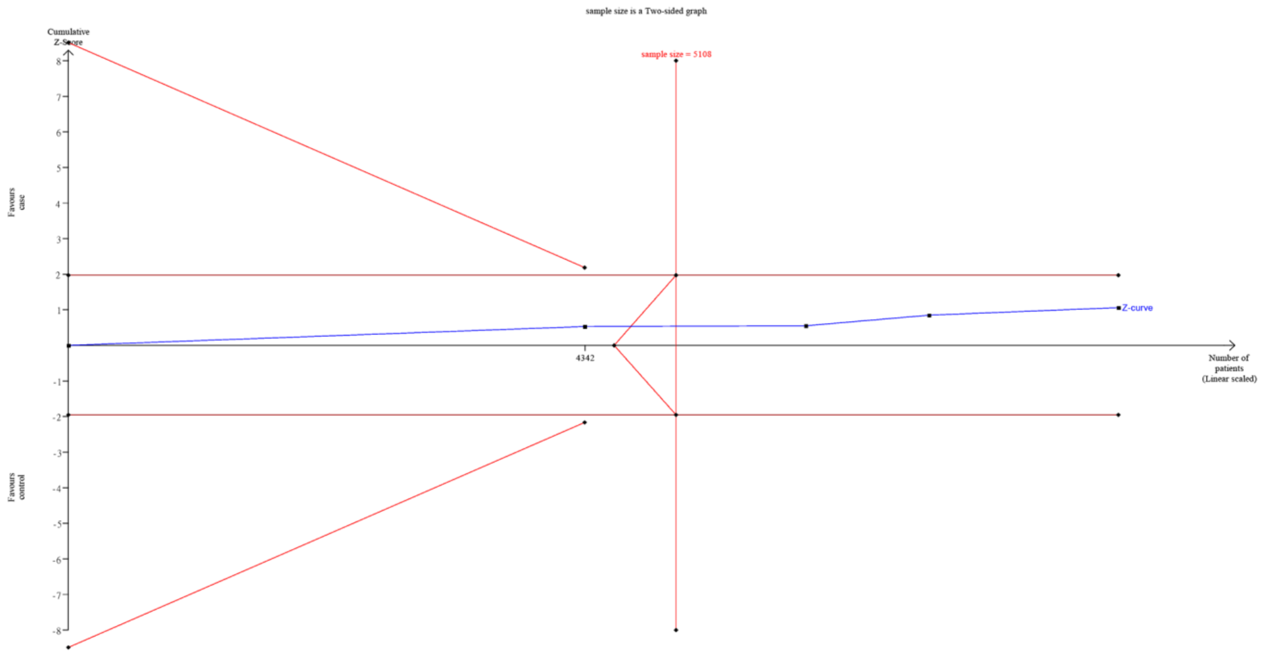

Supplementary Figure 25. Trial sequential analysis of the association between IL-6 rs1800795 polymorphism and the risk of osteoarthritis in Caucasian.

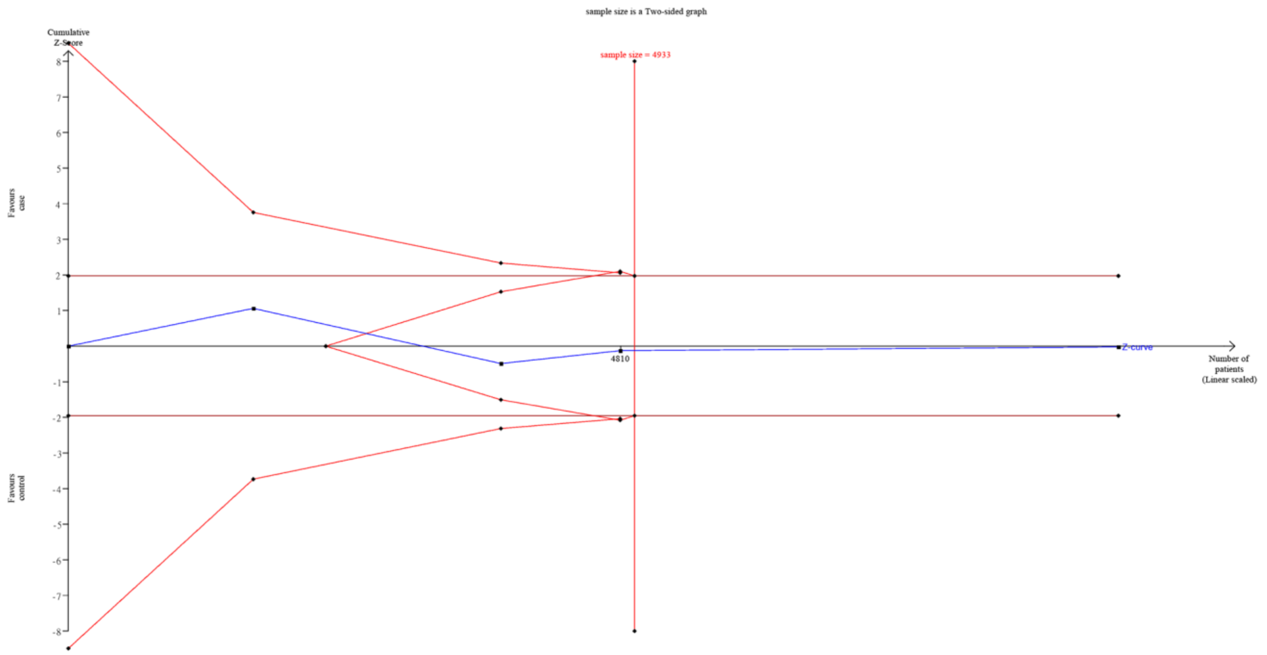

Supplementary Figure 26. Trial sequential analysis of the association between IL-6 rs1800797 polymorphism and the risk of osteoarthritis in Caucasian.

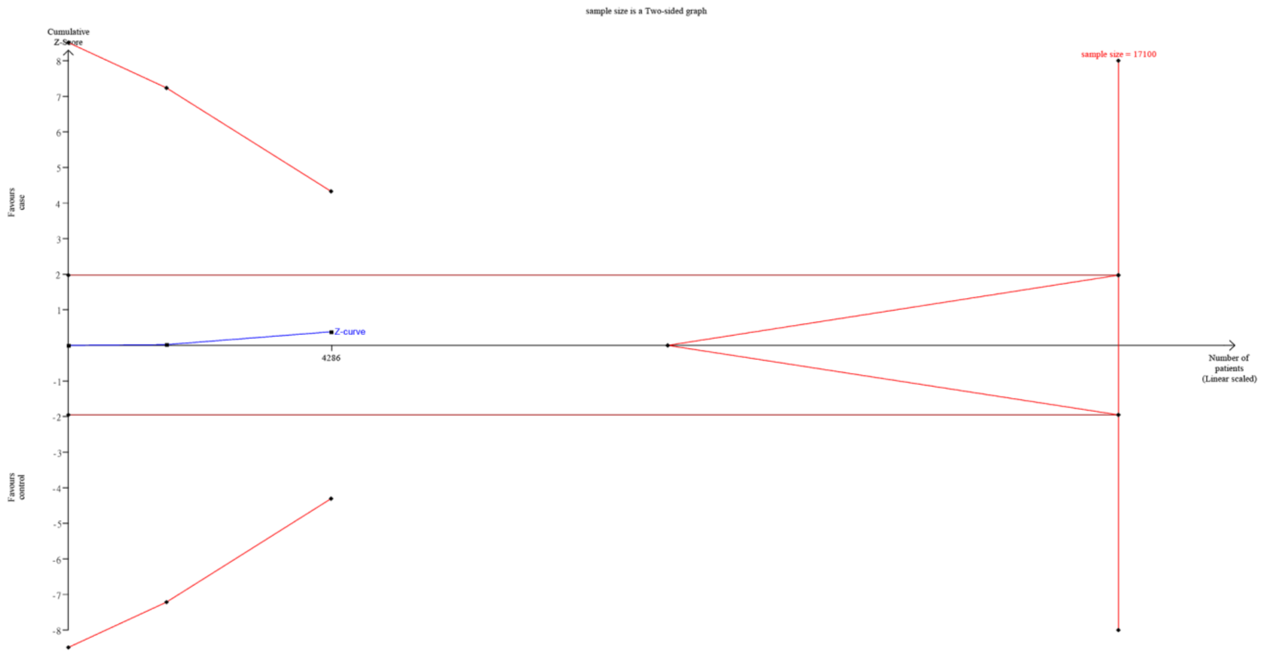

Supplementary Figure 27. Trial sequential analysis of the association between LRCH1 rs912428 polymorphism and the risk of osteoarthritis in Asian.

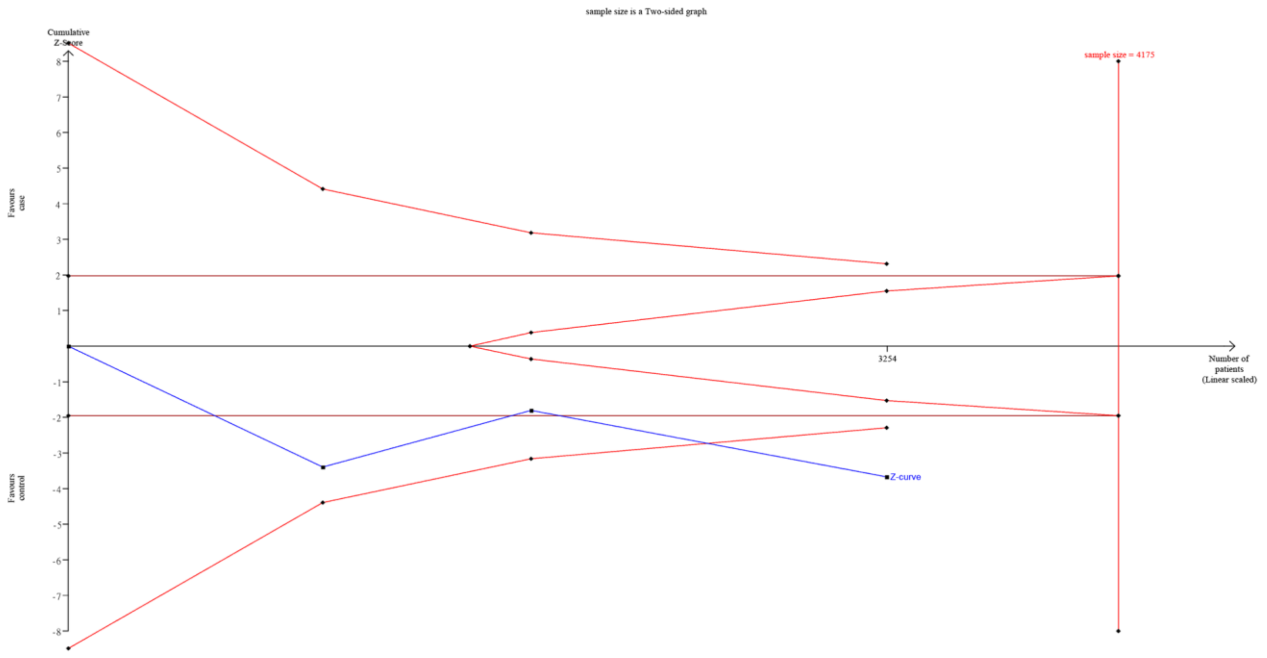

Supplementary Figure 28. Trial sequential analysis of the association between MMP-1 rs1799750 polymorphism and the risk of osteoarthritis in Asian.

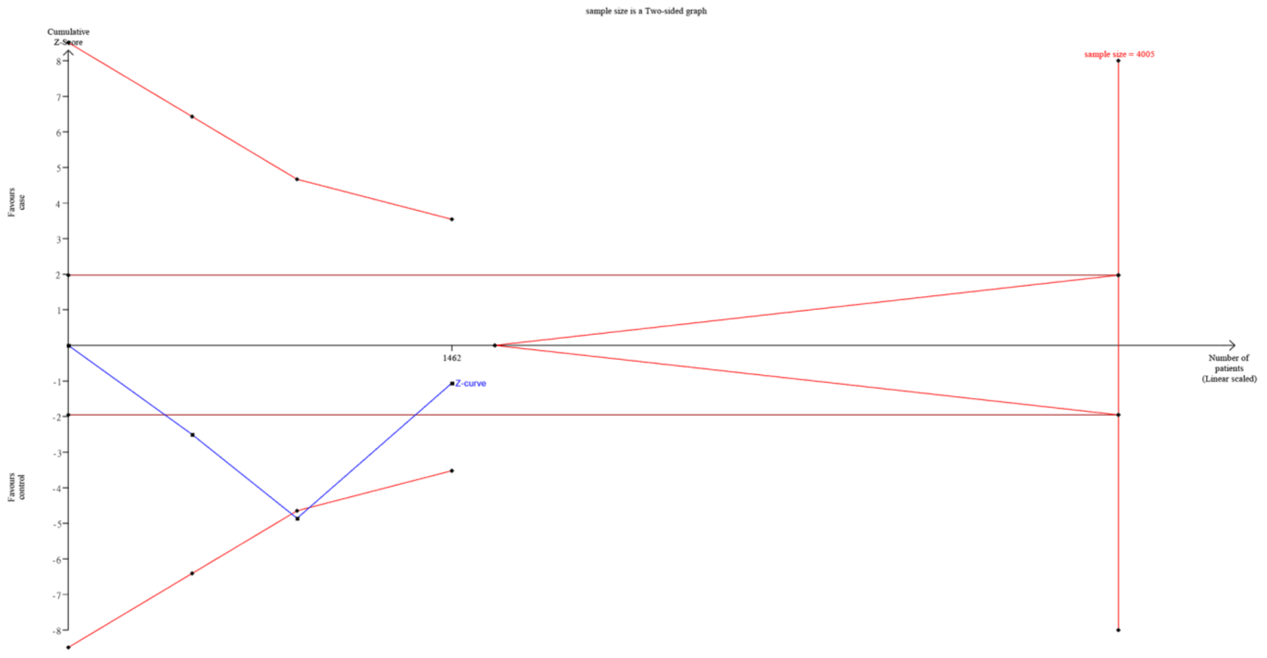

Supplementary Figure 29. Trial sequential analysis of the association between MMP-1 rs1799750 polymorphism and the risk of osteoarthritis in Caucasian.

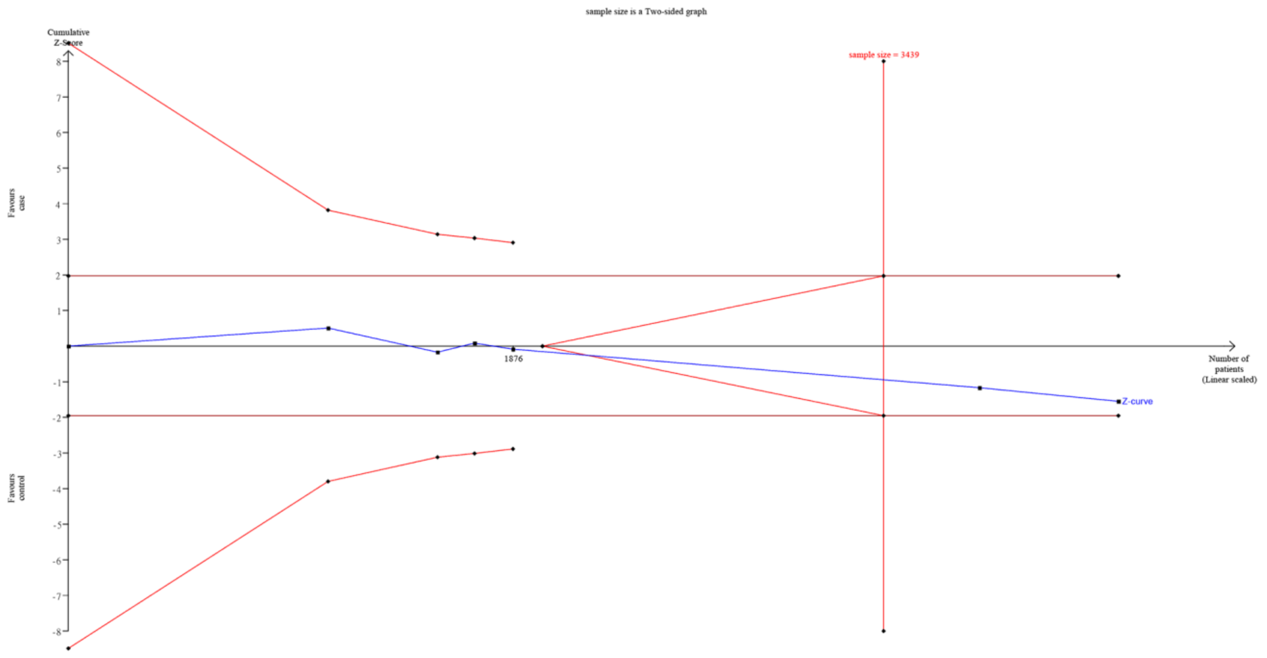

Supplementary Figure 30. Trial sequential analysis of the association between ESR1 rs2234693 polymorphism and the risk of osteoarthritis in Asian.

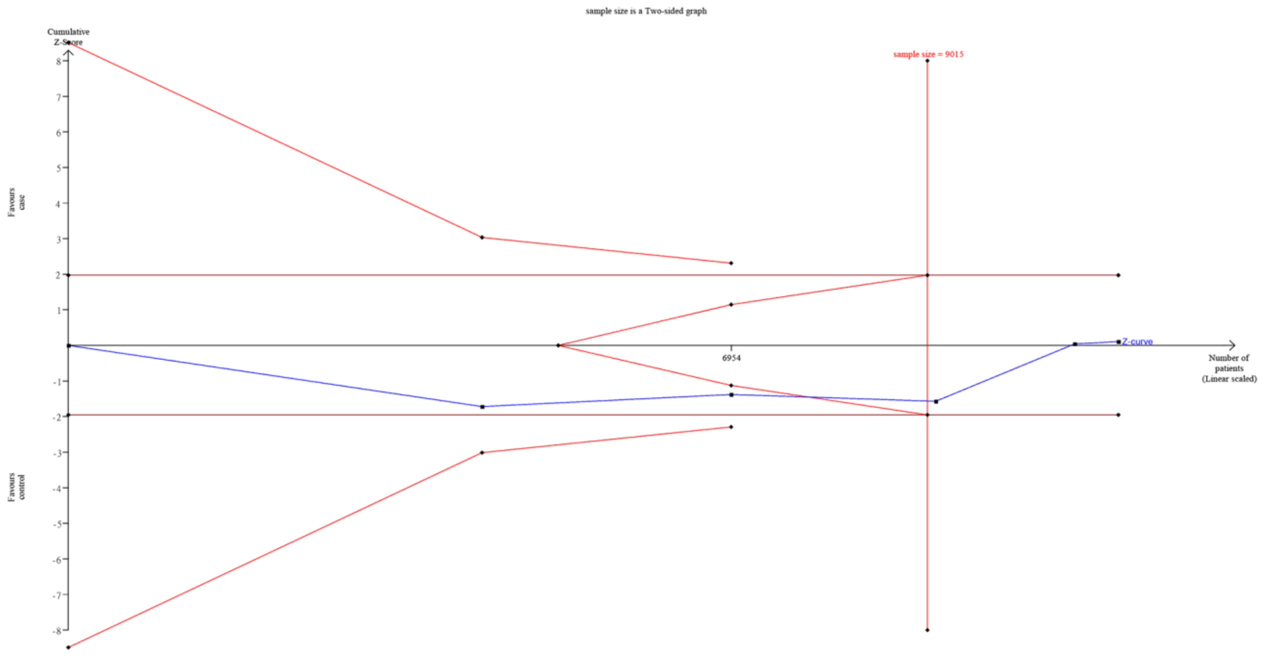

Supplementary Figure 31. Trial sequential analysis of the association between ESR1 rs2234693 polymorphism and the risk of osteoarthritis in Caucasian.

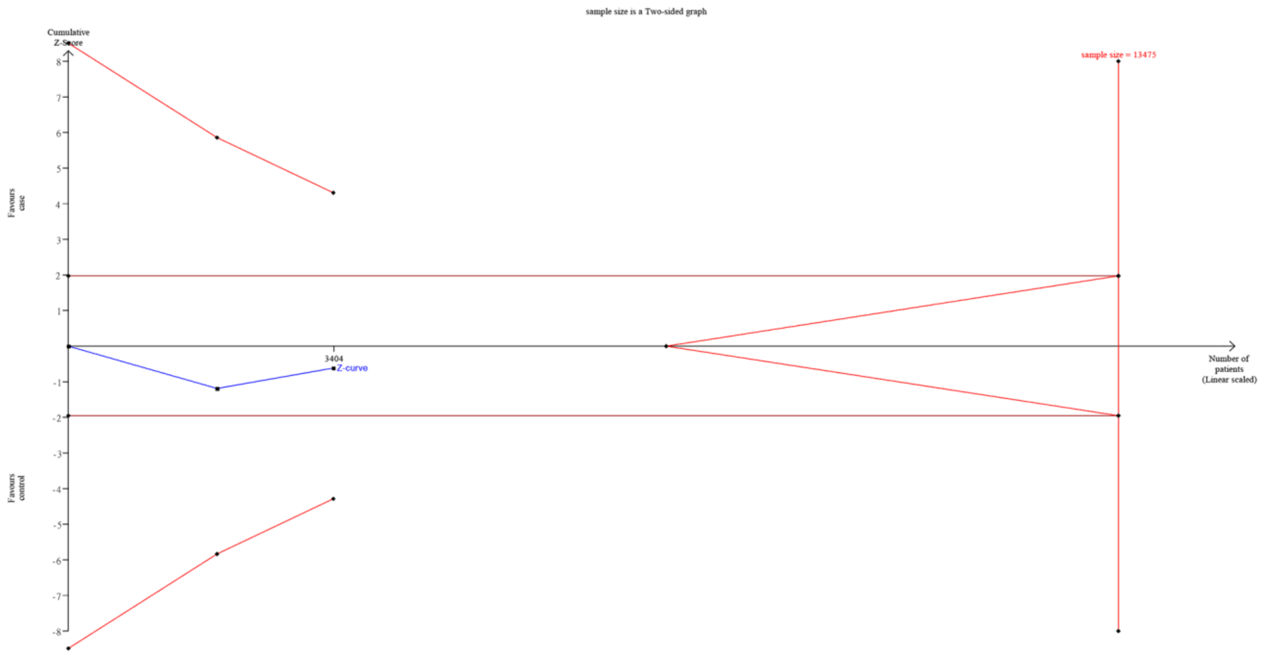

Supplementary Figure 32. Trial sequential analysis of the association between RHOB rs585017 polymorphism and the risk of osteoarthritis in Asian.

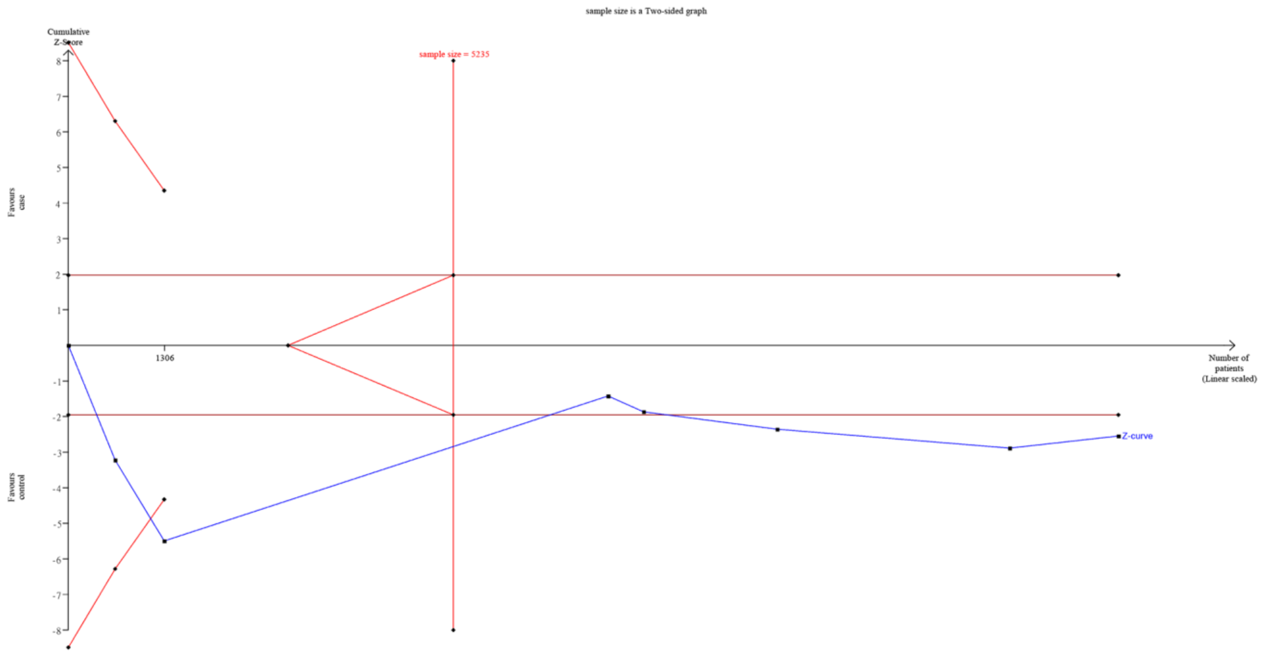

Supplementary Figure 33. Trial sequential analysis of the association between SMAD3 rs12901499 polymorphism and the risk of osteoarthritis in Asian.

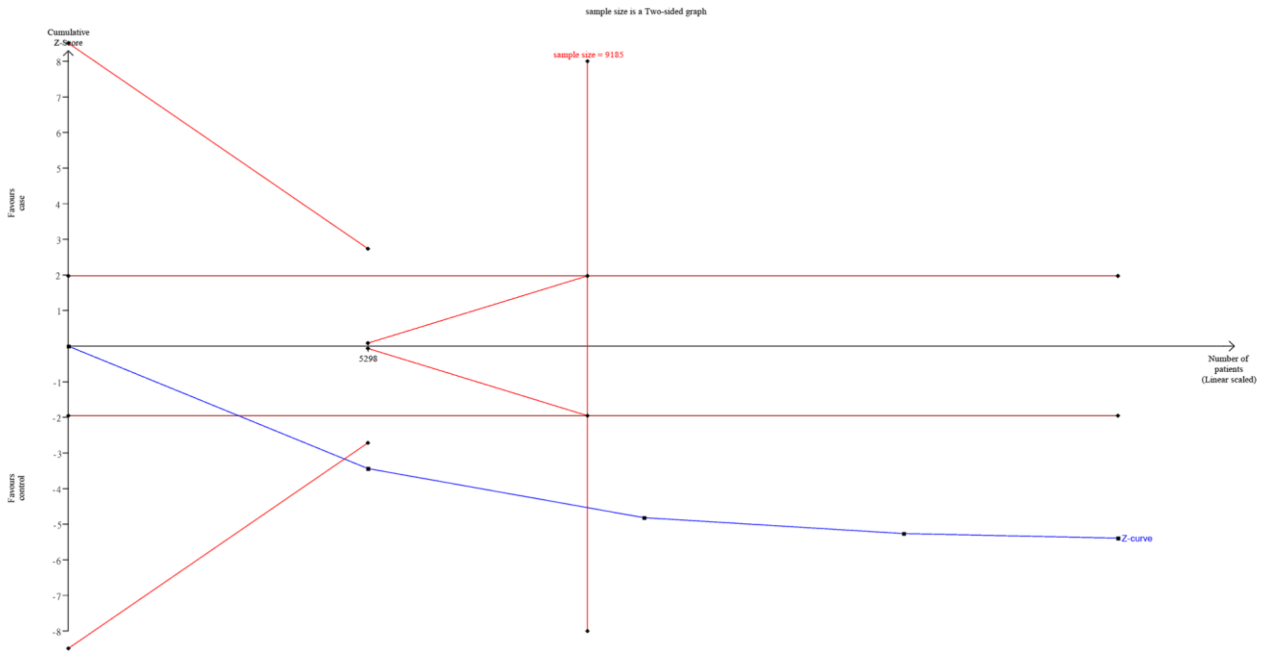

Supplementary Figure 34. Trial sequential analysis of the association between SMAD3 rs12901499 polymorphism and the risk of osteoarthritis in Caucasian.

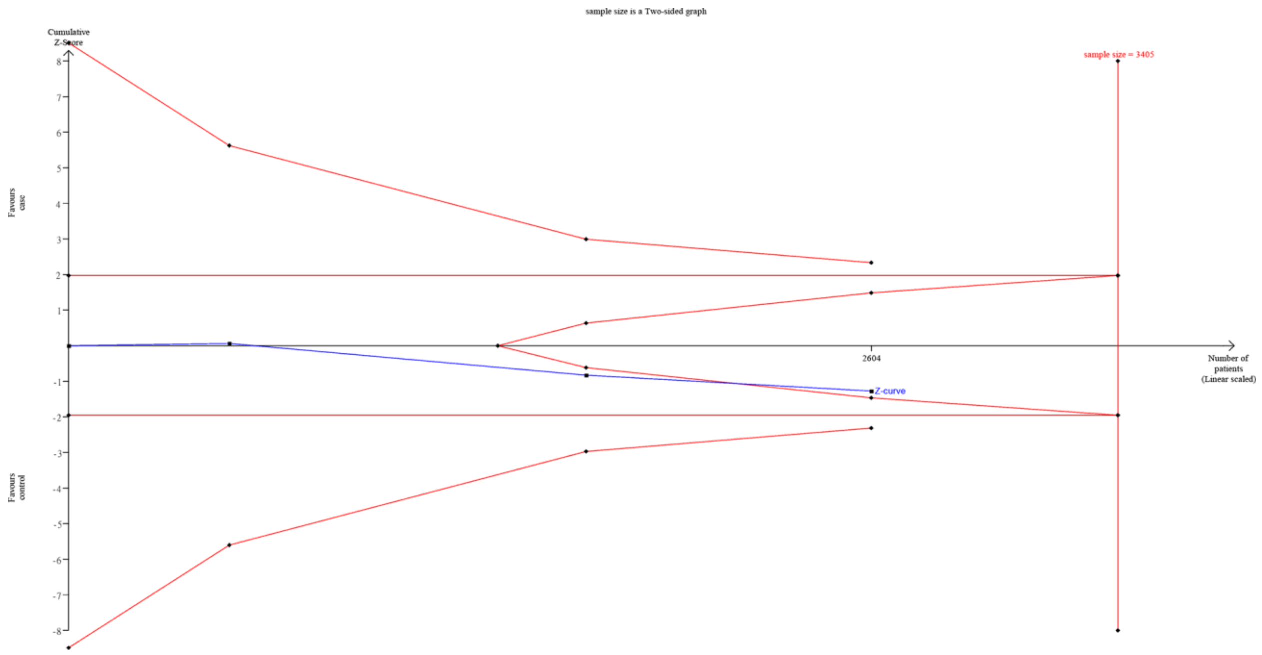

Supplementary Figure 35. Trial sequential analysis of the association between VDR rs731236 polymorphism and the risk of osteoarthritis in Asian.

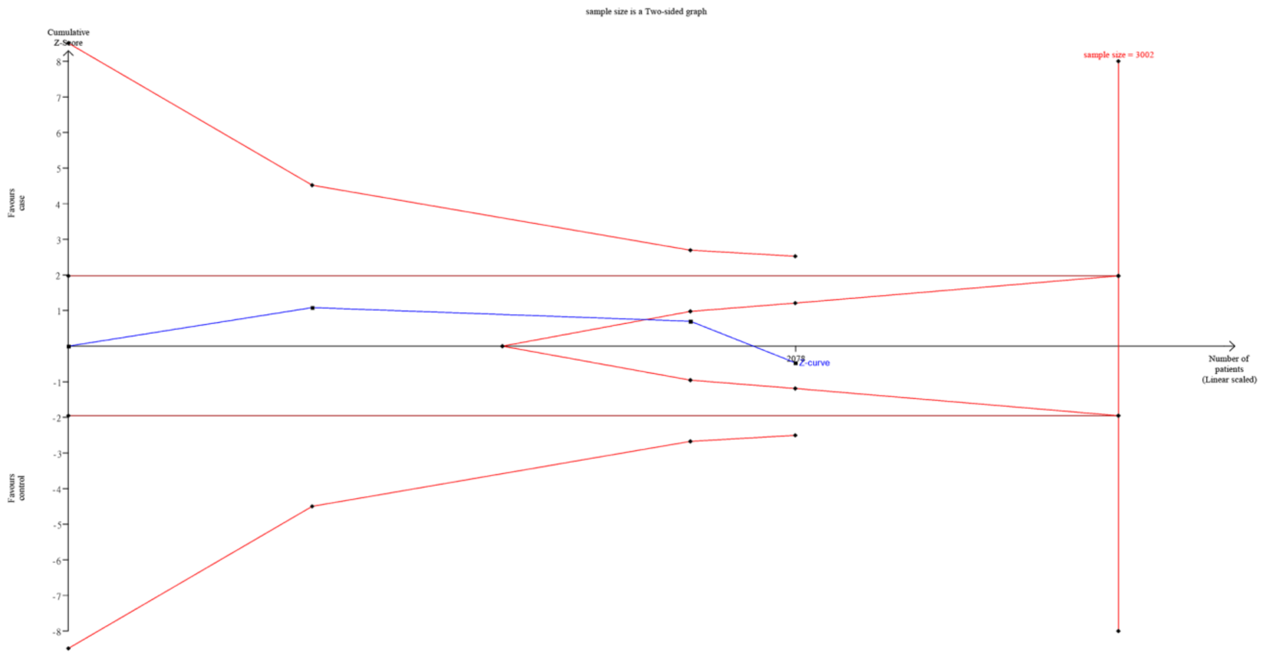

Supplementary Figure 36. Trial sequential analysis of the association between VDR rs731236 polymorphism and the risk of osteoarthritis in Caucasian.

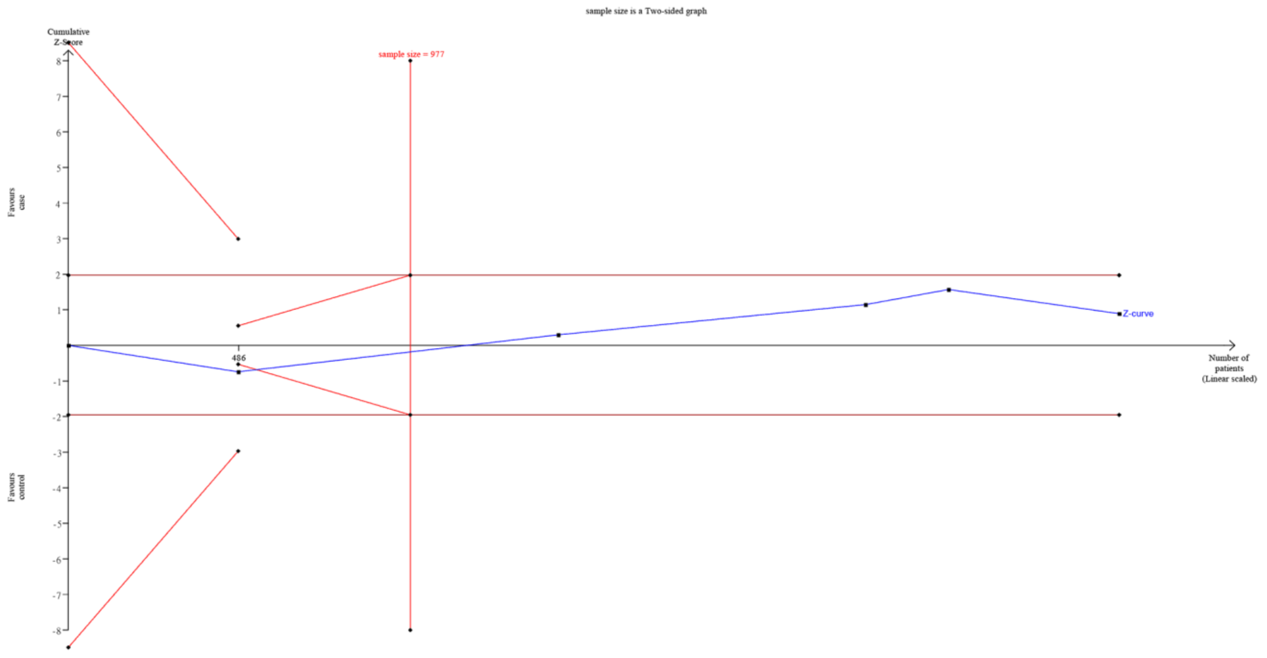

Supplementary Figure 37. Trial sequential analysis of the association between TGF-β rs1982073 polymorphism and the risk of osteoarthritis in Asian.

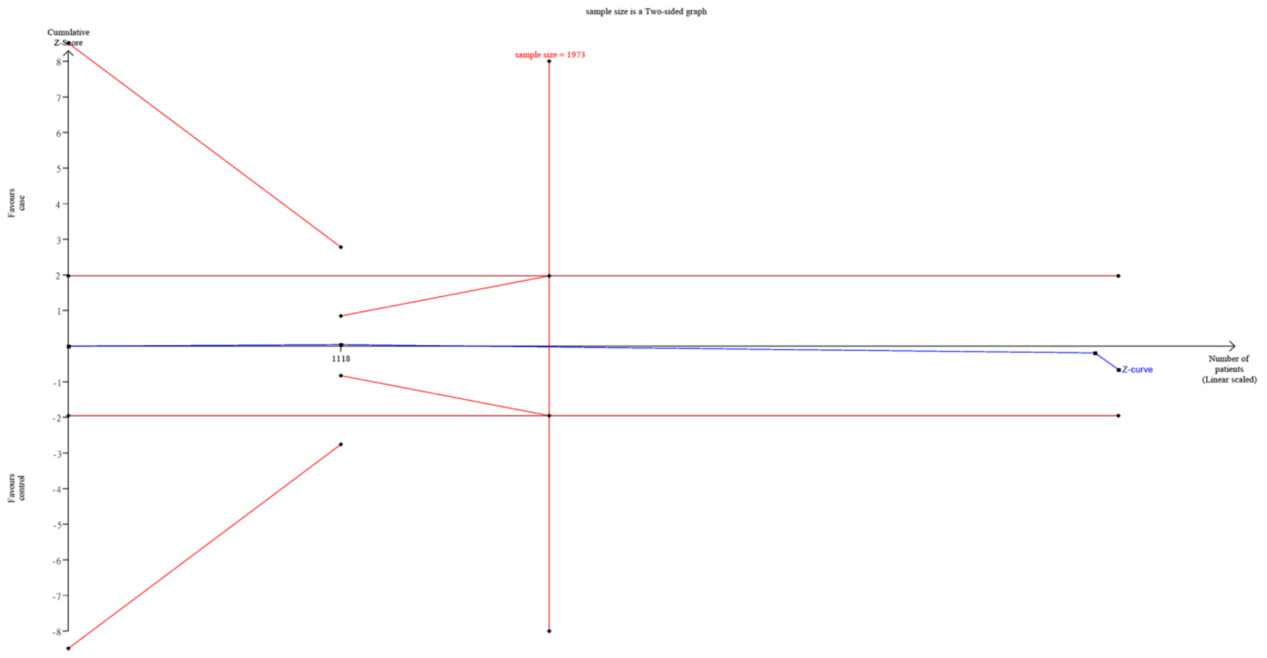

Supplementary Figure 38. Trial sequential analysis of the association between TGF-β rs1982073 polymorphism and the risk of osteoarthritis in Caucasian.

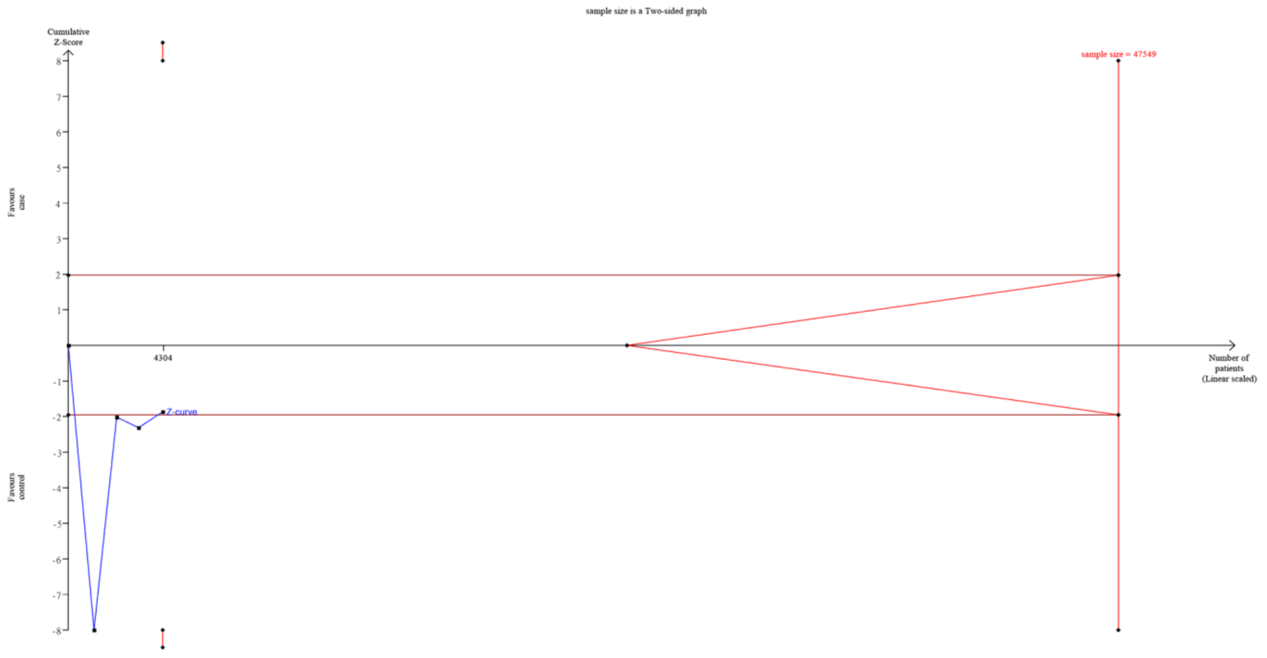

Supplementary Figure 39. Trial sequential analysis of the association between TNF-αrs1800629 polymorphism and the risk of osteoarthritis in Asian.

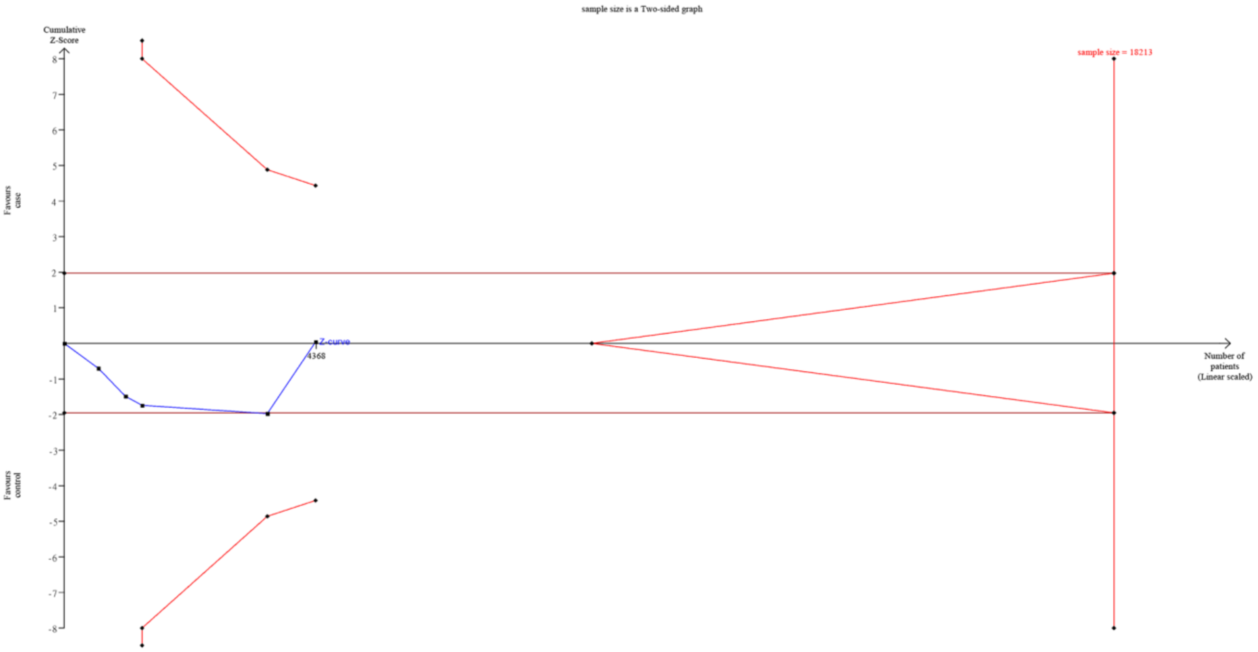

Supplementary Figure 40. Trial sequential analysis of the association between TNF-αrs1800629 polymorphism and the risk of osteoarthritis in Caucasian.

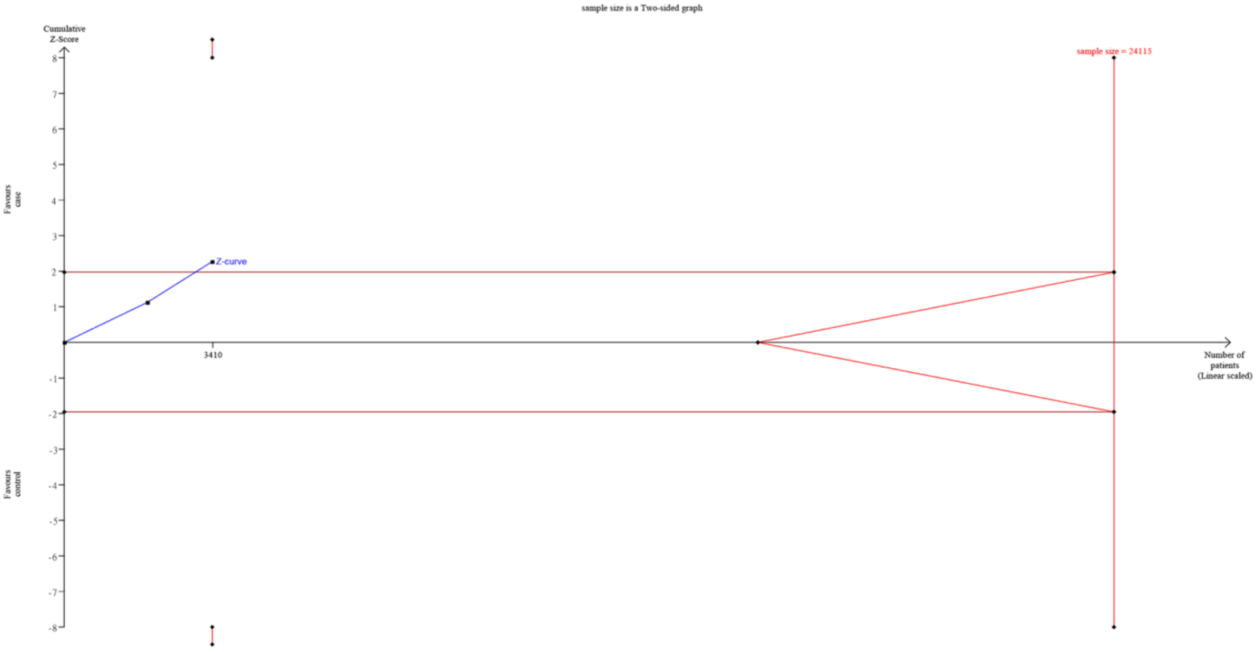

Supplementary Figure 41. Trial sequential analysis of the association between TXNDC3 rs4720262 polymorphism and the risk of osteoarthritis in Asian.

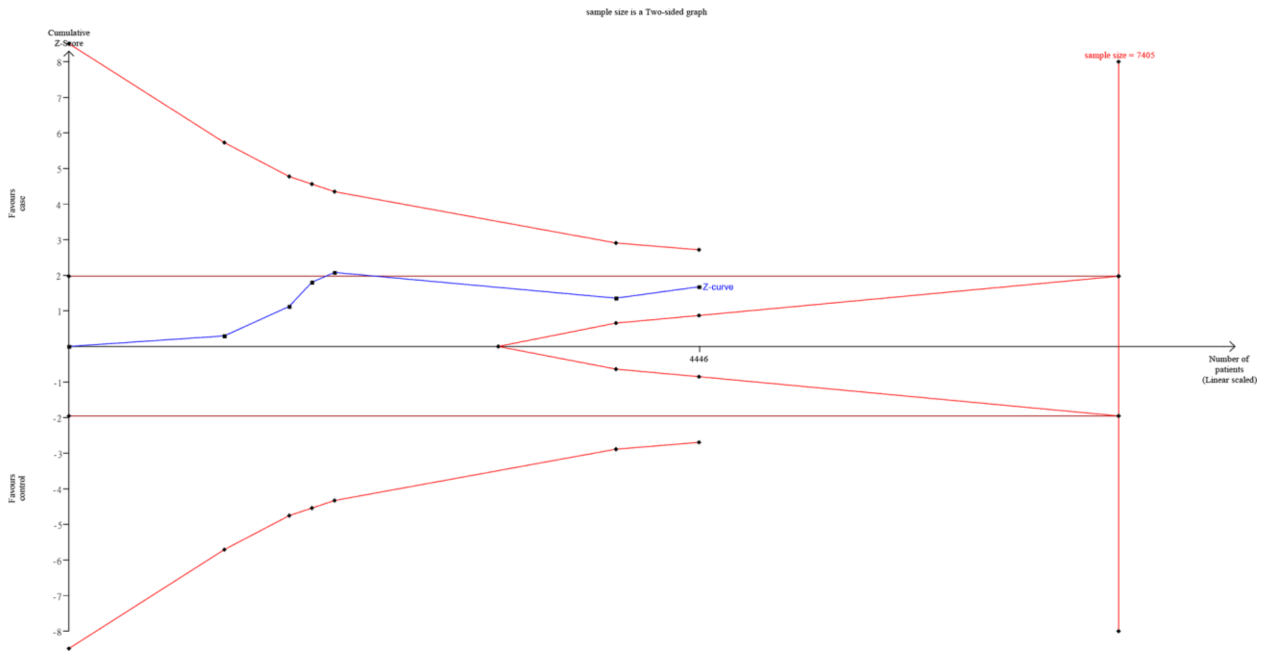

Supplementary Figure 42. Trial sequential analysis of the association between ESR1 rs9340799 polymorphism and the risk of osteoarthritis in Asian.

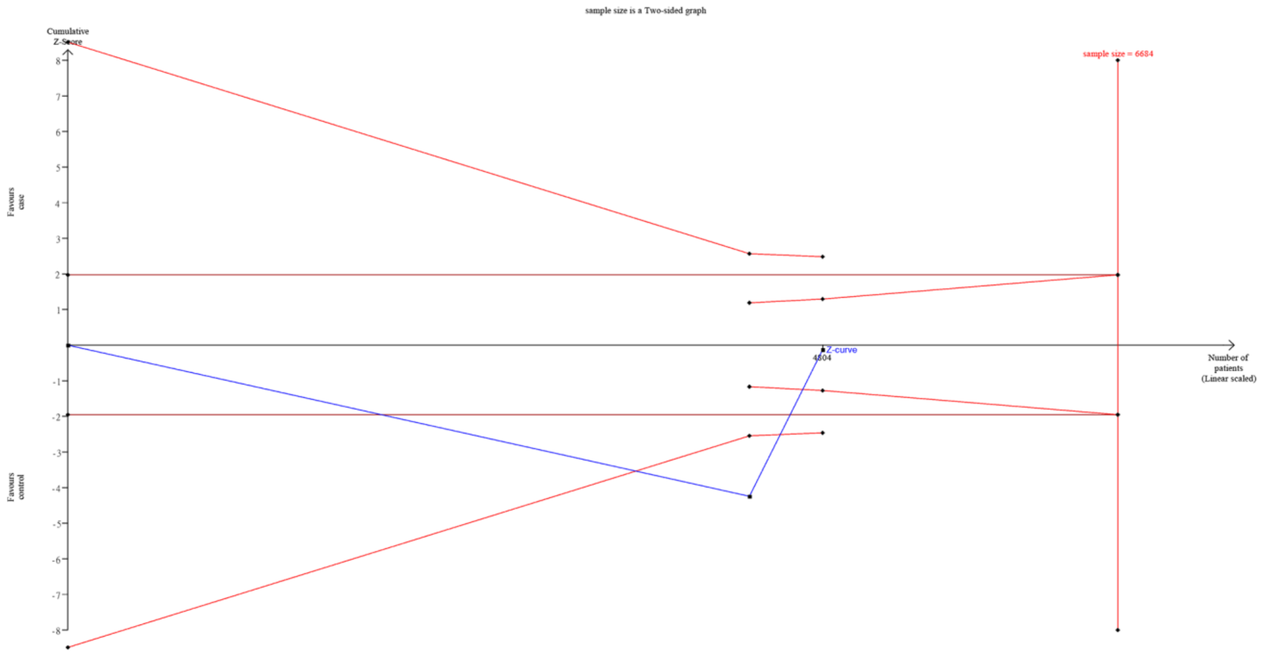

Supplementary Figure 43. Trial sequential analysis of the association between ESR1 rs9340799 polymorphism and the risk of osteoarthritis in Caucasian.
