# Supplementary material for: Decisive gene strategy on osteoarthritis: a comprehensive whole-literature based approach for conclusive gene targets
Source: Aging (Albany NY). 2024 Sep 6;16(17):12346–78. doi: 10.18632/aging.206094 (PMC11424587; doi:10.18632/aging.206094)
Supplement: Supplementary Table 1 [file aging-16-206094-s002.pdf]

SUPPLEMENTARY TABLES

Supplementary Table 1. Search strategies.

**Relevant text of polymorphisms**
1. genetic
2. genetic polymorphism
3. SNP
4. single nucleotide polymorphism
5. polymorphism
6. 1 or 2 or 3 or 4 or 5
**Relevant text of osteoporosis**
7. osteoarthritis
8. osteoarthritides
9. osteoarthrosis
10. osteoarthroses
11. arthritis, degenerative
12. arthritides, degenerative
13. degenerative arthritides
14. degenerative arthritis
15. arthrosis
16. arthroses
17. osteoarthrosis deformans
18. 7 or 8 or 9 or 10 or 11 or 12 or 13 or 14 or 15 or 16 or 17
**Relevant text of meta**
19. meta-analysis
20. meta
21. 19 or 20
**Combined (Final strategy)**
22. 6 and 18 and 21

MeSH Browser: http://www.nlm.nih.gov/mesh/MBrowser.html
PubMed: http://www.ncbi.nlm.nih.gov/pubmed
Cochrane Library: http://www.thecochranelibrary.com
Embase: https://www.embase.com
