# Supplementary material for: Decisive gene strategy on osteoarthritis: a comprehensive whole-literature based approach for conclusive gene targets
Source: Aging (Albany NY). 2024 Sep 6;16(17):12346–78. doi: 10.18632/aging.206094 (PMC11424587; doi:10.18632/aging.206094)
Supplement: Supplementary Table 2 [file aging-16-206094-s003.docx]

| **Supplementary Table 2. Association of candidate gene SNPs with OA in Caucasian.** | | | | | | | | | | | | |
| --- | --- | --- | --- | --- | --- | --- | --- | --- | --- | --- | --- | --- |
|  | Gene | Rs number | Numbers of article | Major/  Minor | MAF* | Result of TSA | Case/Control | Test of association | | Test of heterogeneity | |  |
|  |  |  |  |  |  |  |  | OR  (95%CI) | p-value | I^2^ | p-value |  |
| 1 | ESR1 | rs9340799 | 2 | A/G | 31% | Determine the mutation is not significantly associated with osteoarthritis | 1598/804 | 1.05  (0.56-1.95) | 0.889 | 87% | 0.005 |  |
| 2 | ESR1 | rs2234693 | 5 | T/C | 42% | Determine the mutation is not significantly associated with osteoarthritis | 2569/2941 | 0.99  (0.87-1.13) | 0.917 | 56% | 0.060 |  |
| 3 | CYP19A1 | rs700518 | 4 | T/C | 47% | Determine the mutation is not significantly associated with osteoarthritis | 1304/1706 | 0.94  (0.80-1.11) | 0.474 | 55% | 0.082 |  |
| 4 | ESR2 | rs1256031 | 7 | G/A | 43% | Determine the mutation is not significantly associated with osteoarthritis | 2364/6773 | 1.07  (0.98-1.17) | 0.121 | 25% | 0.236 |  |
| 5 | DVWA | rs7639618 | 7 | C/T | 16% | Determine the mutation is not significantly associated with osteoarthritis | 2178/3414 | 0.98  (0.84-1.14) | 0.761 | 46% | 0.082 |  |
| 6 | GDF5 | rs143383 | 11 | A/G | 37% | Determine the mutation is not significantly associated with osteoarthritis | 8131/10142 | 0.93  (0.82-1.06) | 0.285 | 87% | 0.001 |  |
| 7 | VDR | rs731236 | 3 | A/G | 40% | Determine the mutation is not significantly associated with osteoarthritis | 273/766 | 1.10  (0.74-1.65) | 0.629 | 69% | 0.037 |  |
| 8 | TGF-β | rs1982073 | 3 | A/G | 38% | Determine the mutation is not significantly associated with osteoarthritis | 1684/471 | 1.09  (0.84-1.41) | 0.508 | 44% | 0.168 |  |
| 9 | IL-6 | rs1800795 | 4 | C/G | 42% | Determine the mutation is not significantly associated with osteoarthritis | 1904/2511 | 0.95  (0.86-1.05) | 0.293 | 0% | 0.836 |  |
| 10 |  | rs1800797 | 4 | A/G | 41% | Determine the mutation is not significantly associated with osteoarthritis | 1914/2026 | 1.00  (0.81-1.24) | 0.979 | 72% | 0.012 |  |
| 11 | DIO3 | rs945006 | 4 | T/G | 10% | Determine the mutation is not significantly associated with osteoarthritis | 1073/2106 | 0.80  (0.65-0.99) | 0.135 | 24% | 0.267 |  |
| 12 | EDG2 | rs10980705 | 4 | C/T | 24% | Determine the mutation is not significantly associated with osteoarthritis | 1501/2521 | 1.20  (1.12-1.29) | 0.237 | 80% | 0.001 |  |
| 13 | FRZB | rs288326 | 4 | G/A | 12% | Determine the mutation is not significantly associated with osteoarthritis | 2660/3040 | 1.00  (0.84-1.18) | 0.962 | 39% | 0.18 |  |
| 14 |  | rs7775 | 4 | G/C | 9% | Determine the mutation is not significantly associated with osteoarthritis | 2706/3109 | 1.08  (0.94-1.23) | 0.295 | 0% | 0.94 |  |
| 15 | CALM1 | rs12885713 | 2 | C/T | 34% | Determine the mutation is not significantly associated with osteoarthritis | 1574/1438 | 1.08  (0.97-1.19) | 0.164 | 0% | 0.410 |  |
| 16 | FAS | rs1800682 | 5 | A/G | 54% | Determine the mutation is not significantly associated with osteoarthritis | 571/529 | 1.07  (0.90-1.28) | 0.452 | 0% | 0.73 |  |
| 17 | ADAMTS5 | rs226794 | 3 | A/G | 73% | Determine the mutation is not significantly associated with osteoarthritis | 1719/971 | 0.95  (0.84-1.08) | 0.423 | 0% | 0.50 |  |
| 18 | TNF-α | rs1800629 | 2 | G/A | 13% | Still need to accumulate 14000 samples to determine | 817/1426 | 1.74  (0.95-3.19) | 0.071 | 0% | 0.359 |  |
| 19 | ADAM12 | rs3740199 | 6 | C/G | 46% | Still need to accumulate 4000 samples to determine | 163/215 | 0.90  (0.73-1.11) | 0.327 | 58% | 0.068 |  |
| 20 | VDR | rs1544410 | 2 | C/T | 40% | Still need to accumulate 1000 samples to determine | 391/475 | 1.02  (0.61-1.72) | 0.934 | 77% | 0.038 |  |
| 21 | COX2 | rs20417 | 2 | C/G | 15% | Still need to accumulate 25000 samples to determine | 677/696 | 0.91  (0.33-2.48) | 0.848 | 92% | 3.0E-4 |  |
| 22 | MMP-1 | rs1799750 | 3 | C/- | 38% | Still need to accumulate 1272 samples to determine | 411/320 | 1.46  (0.73-2.92) | 0.288 | 90% | 0.01 |  |
| 23 | SMAD3 | rs12901499 | 4 | G/A | 46% | Determine the mutation is significantly associated with osteoarthritis (Risk) | 3170/6114 | 1.20  (1.12-1.29) | 0.001 | 0% | 0.974 |  |

*MAF：Minor allele frequency, data from http://grch37.ensembl.org/index.html
