# Supplementary material for: Decisive gene strategy on osteoarthritis: a comprehensive whole-literature based approach for conclusive gene targets
Source: Aging (Albany NY). 2024 Sep 6;16(17):12346–78. doi: 10.18632/aging.206094 (PMC11424587; doi:10.18632/aging.206094)
Supplement: Supplementary Table 3 [file aging-16-206094-s004.docx]

| **Supplementary Table 3. Association of candidate gene SNPs with OA in Asian.** | | | | | | | | | | | |
| --- | --- | --- | --- | --- | --- | --- | --- | --- | --- | --- | --- |
|  | Gene | Rs number | Numbers of article | Major/minor | MAF* | Result of TSA | Case/Control | Test of association | | Test of heterogeneity | |
|  |  |  |  |  |  |  |  | OR(95%CI) | p-value | I^2^ | p-value |
| 1 | ESR1 | rs2234693 | 6 | T/C | 40% | Determine the mutation is not significantly associated with osteoarthritis | 852/1363 | 1.11  (0.97-1.25) | 0.119 | 0% | 0.687 |
| 2 | CYP19A1 | rs700518 | 2 | T/C | 44% | Determine the mutation is not significantly associated with osteoarthritis | 349/347 | 1.11  (0.80-1.54) | 0.538 | 54% | 0.140 |
| 3 | ADAM12 | rs3740199 | 6 | C/G | 48% | Determine the mutation is not significantly associated with osteoarthritis | 925/1937 | 0.90  (0.73-1.11) | 0.327 | 58% | 0.068 |
| 4 | ACE I/D | rs4340 | 6 | I/D | - | Determine the mutation is not significantly associated with osteoarthritis | 1165/1029 | - | - | - | - |
| 5 | VDR | rs731236 | 3 | A/G | 7% | Determine the mutation is not significantly associated with osteoarthritis | 700/602 | 1.3  (0.87-1.95) | 0.202 | 19% | 0.291 |
| 6 | TGF-β | rs1982073 | 5 | A/G | 55% | Determine the mutation is not significantly associated with osteoarthritis | 921/578 | 0.93  (0.78-1.10) | 0.380 | 17% | 0.308 |
| 7 | FAS | rs1800682 | 5 | A/G | 49% | Determine the mutation is not significantly associated with osteoarthritis | 1258/1086 | 0.90  (0.80-1.02) | 0.086 | 0% | 0.846 |
| 8 | ADAMTS5 | rs226794 | 3 | A/G | 50% | Determine the mutation is not significantly associated with osteoarthritis | 1051/997 | 0.95  (0.84-1.08) | 0.423 | 0% | 0.506 |
| 9 | ESR1 | rs9340799 | 6 | A/G | 19% | Still need to accumulate 1087 samples to determine | 852/1371 | 0.73  (0.50-1.06) | 0.093 | 81% | 1.0E-4 |
| 10 | TNF-α | rs1800629 | 4 | G/A | 6% | Still need to accumulate 40000 samples to determine | 950/1202 | 2.40  (0.96-6.02) | 0.061 | 97% | 0.001 |
| 11 | RHOB | rs585017 | 2 | A/G | 9% | Still need to accumulate 5035 samples to determine | 845/857 | 1.15  (0.75-1.75) | 0.532 | 23% | 0.255 |
| 12 | TXNDC3 | rs4720262 | 2 | C/T | 12% | Still need to accumulate 10352 samples to determine | 845/860 | 0.78  (0.62-0.97) | 0.023 | 0% | 0.515 |
| 13 | LRCH1 | rs912428 | 2 | A/G | 7% | Still need to accumulate 6407 samples to determine | 1038/1105 | 0.96  (0.77-1.20) | 0.716 | 0% | 0.788 |
| 14 | CALM1 | rs12885713 | 2 | C/T | 20% | Still need to accumulate1628 samples to determine | 517/585 | 1.20  (0.89-1.63) | 0.227 | 54% | 0.140 |
| 15 | ESR1 | rs2228480 | 3 | G/A | 17% | Determine the mutation is significantly associated with osteoarthritis (Risk) | 331/619 | 1.35  (1.08-1.69) | 0.007 | 0% | 0.445 |
| 16 | SMAD3 | rs12901499 | 7 | G/A | 48% | Determine the mutation is significantly associated with osteoarthritis (Risk) | 2168/4966 | 1.34  (1.07-1.69) | 0.010 | 87% | 0.001 |
| 17 | MMP-1 | rs1799750 | 3 | CC/C | 33% | Determine the mutation is significantly associated with osteoarthritis (Risk) | 834/793 | 1.43  (1.18-1.74) | 0.0002 | 43% | 0.173 |
| 18 | DVWA | rs7639618 | 6 | C/T | 48% | Determine the mutation is significantly associated with osteoarthritis (Protective) | 4629/4371 | 0.78  (0.67-0.90) | 0.0007 | 79% | 2.0E-4 |
| 19 | GDF5 | rs143383 | 4 | A/G | 29% | Determine the mutation is significantly associated with osteoarthritis (Protective) | 1780/2345 | 0.74  (0.67-0.81) | <0.001 | 0% | 0.615 |
| 20 | VDR | rs7975232 | 4 | C/A | 29% | Determine the mutation is significantly associated with osteoarthritis (Protective) | 590/1443 | 0.56  (0.35-0.90) | 0.015 | 81% | 0.001 |

*MAF：Minor allele frequency, data from http://grch37.ensembl.org/index.html
